# Multifunctional PHC Bandage for Accelerated Wound Healing in Movable Parts

**DOI:** 10.1002/EXP.20230176

**Published:** 2025-03-06

**Authors:** Liqi Wei, Xin Liu, Yuanqiang Li, Yu Han, Yiping Ren, Tianshu Zou, Pengcheng Yu, Yining Chen, Biao Zhang, Zixuan Wang, Jingyi Jiang, Yumi Kim, Rui Chen, Yan Cheng, Hongxia Ma

**Affiliations:** ^1^ Engineering Research Center of the Chinese Ministry of Education for Bioreactor and Pharmaceutical Development College of Life Science Jilin Agricultural University Changchun China; ^2^ Jilin Provincial Key Laboratory of Human Health Status Identification and Function Enhancement School of Materials Science and Engineering Changchun University Changchun China; ^3^ Department of Biomedical Engineering Ulsan National Institute of Science and Technology (UNIST) Ulsan Republic of Korea

**Keywords:** bacterial inactivation, cell migration and proliferation, piezoelectric material, wound dressing

## Abstract

Wound healing in movable parts poses challenges owing to frequent activities, leading to delayed recovery and heightened susceptibility to bacterial infections and inflammation. Although hydrogel‐based dressings have been explored, their therapeutic effectiveness is limited by poor resistance to stimuli and low mechanical strength. Here, we present a novel multifunctional PHC bandage that prevents bacterial infection and capitalizes on the inherent mobility of the affected area to expedite the wound‐healing process. A PHC bandage was fabricated by incorporating photothermal copper bismuth sulfide (Cu_3_BiS_3_) nanomaterials into piezoelectric and pyroelectric polyvinylidene fluoride (PVDF). Upon exposure to alternating near‐infrared light, the embedded Cu_3_BiS_3_ generated localized heat, activated PVDF, and induced the production of abundant reactive oxygen species for bacterial inactivation. Furthermore, continuous movement of the wound area triggers the PVDF to generate a sustained electrical field, promoting cell migration and proliferation to facilitate wound healing. The wound healing rate of PHC was 13.17 ± 2.09% higher than medical gauze. The robust encapsulation of PVDF ensured secure containment of the loaded Cu_3_BiS_3_ nanoparticles, improving the biocompatibility and sustainable utilization of this innovative wound dressing. This innovative design offers a promising and effective solution for improving wound healing in movable parts, potentially revolutionizing wound care technology.

## Graphical abstract

Multifunctional and reusable PHC wound dressing was designed for accelerating movable wound healing process. With alternated NIR light irradiation, PHC can produce abundant heat and ROS for bacteria inactivation due to its photothermal and pyroelectric property. With the frequent movement of movable wound, PHC can effectively promote cell migration and proliferation due to its piezoelectric property.

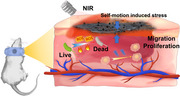

## 1 Introduction

Wounds, especially those located in movable parts, such as the elbows, knees, wrists, and nape, usually undergo delayed and inefficient healing owing to frequent movements in these areas [1]. Without effective treatment, these wounds are prone to secondary injuries during movement, inevitably increasing the risk of bacterial infection and inflammation. Consequently, the wound may undergo repeated stages of inflammation and proliferation, leading to a prolonged healing time and potential disability. Numerous wound dressings, such as gauze [2], hydrogels [3, 4], foams [5], and nanofibers [6, 7], have been developed to address various types of wounds [8, 9]. Wound dressings have been designed with efficient antibacterial ability, excellent stretchability, good tissue adhesion, and self‐healing ability to expedite the wound healing process in movable parts [10, 11]. However, most of these dressings are primarily based on a hydrogel design, which has inherent limitations, such as poor resistance to stimuli and low mechanical strength [12]. Thus, there is a need to develop new dressings that can effectively promote wound healing in movable parts and prevent bacterial infections for further clinical applications. Moreover, it would be highly beneficial if the mobility characteristics of the wound could be utilized to accelerate wound healing.

Piezoelectric materials, which are known to generate an electrical field in response to a mechanical force, have found diverse applications in medical devices [13], bone repair [14, 15], nerve regeneration [16], and even tumor therapy [17]. Electrical stimulation has been demonstrated to influence cell proliferation, differentiation, and regeneration [18, 19]. Notably, a few piezo‐ and pyro‐materials have been employed to promote wound healing by acting on a typical wound (inactive part) via external mechanical forces such as hang‐held massage devices [20] and ultrasonic stimulation [21]. Shi et al. [21] designed flexible piezoelectric poly (vinylidene fluoride‐co‐trifluoroethylene)/barium titanate wound dressings by electrospinning. Ultrasonic stimulation can accelerate the wound‐healing process by generating electric fields. Meng et al. [22] prepared polydopamine@tetragonal barium titanate NPs, which can be excited by near‐infrared (NIR) light to create temperature variations and produce ROS to kill bacteria, owing to the pyroelectric property of barium titanate. The use of external mechanical force for continuous electric field production in clinical settings is cumbersome. Self‐motion can activate piezoelectric materials to produce electric fields. ZnO has been developed for piezoelectric patches [23] and hydrogels [24], which can produce a piezoelectric potential upon mechanical deformation induced by animal motion, thus inducing an electric field at the wound bed to promote wound healing. Considering the frequent movement of movable wounds, we hypothesized that the stress produced by movable wounds could trigger piezoelectric materials to produce electrical fields more efficiently, potentially leading to enhanced cell proliferation and migration. Piezoelectric materials have inherent pyroelectric properties. When these materials are combined with photothermal nanoparticles, they demonstrate the ability to rapidly generate electrons and holes under photoregulation, resulting in the production of reactive oxygen species (ROS). Moreover, the combination of piezoelectric and pyroelectric properties effectively inhibits electron‐hole recombination, a process that can occur in non‐centrosymmetric pyroelectric crystals owing to spontaneous polarization from dipole moments [25]. Consequently, the integration of piezoelectric and pyroelectric materials with photothermal nanoparticles renders them highly promising candidates for the development of multifunctional bandages. These advanced bandages would exhibit antibacterial properties and offer enhanced wound‐healing performance.

**FIGURE 1:**
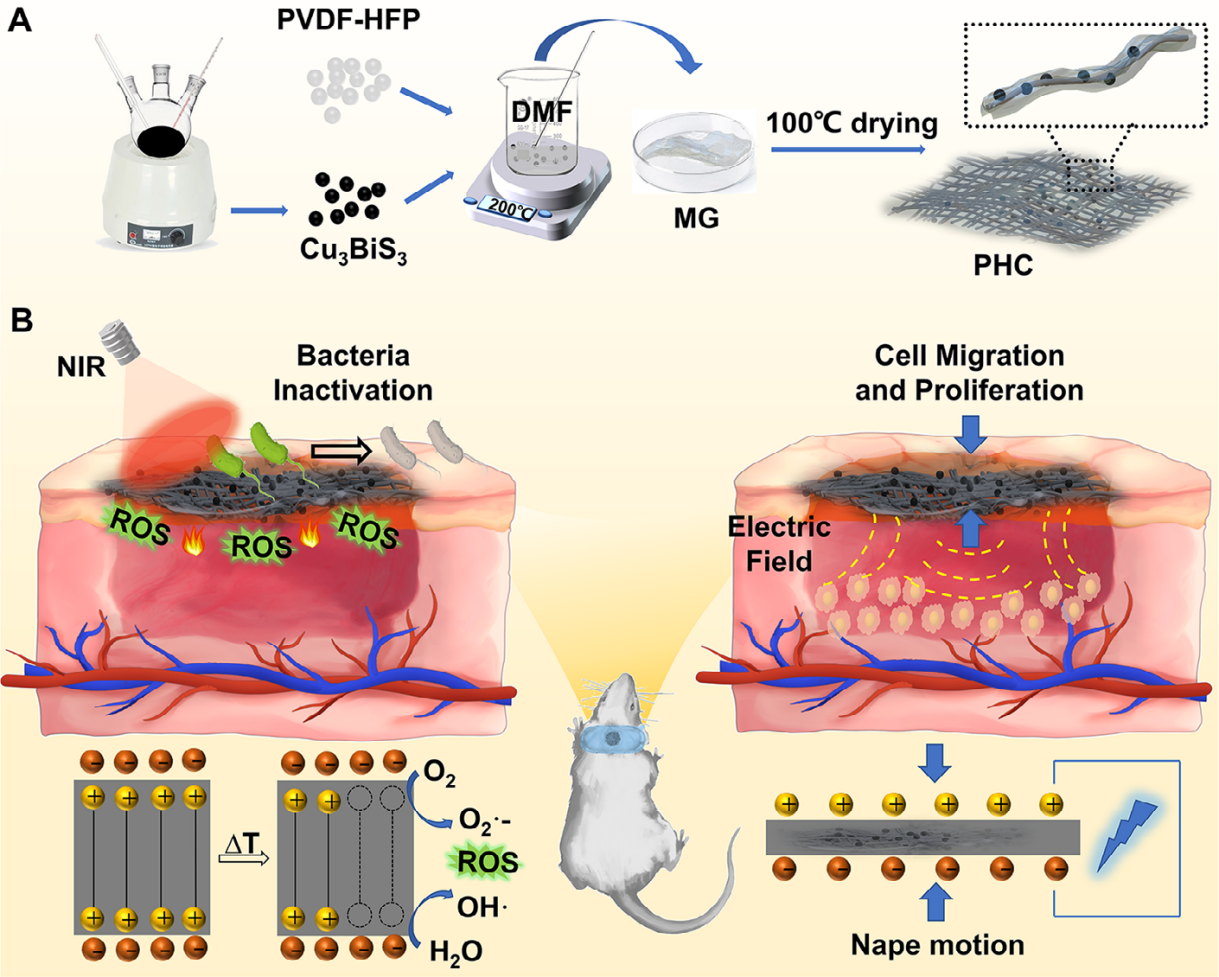
Schematic diagram of fabrication and promoting wound healing performance of PHC wound dressing. (A) The fabrication process of PHC wound dressing; (B) NIR light‐activated antibacterial activity of PHC wound dressing based photothermal and pyro‐generated ROS, as well as cell migration and proliferation abilities based on nape motion.

In this study, we developed a multifunctional (piezoelectric, pyroelectric, and photothermal) bandage to accelerate wound healing in mobile body parts. In this innovative system, we carefully selected the β phase of polyvinylidene fluoride‐co‐hexafluoropropylene (PVDF‐HFP) as the piezoelectric and pyroelectric material, as it has demonstrated remarkable efficiency in generating both electric fields and ROS [26]. Additionally, copper bismuth sulfide (Cu_3_BiS_3_) was chosen as the photoregulated thermal source to activate PVDF‐HFP owing to its excellent NIR‐triggered photothermal performance and cost‐effectiveness. As shown in Figure 1A, Cu_3_BiS_3_ nanoparticles (NPs) fabricated by thermal injection and PVDF‐HFP were first melted in an *N*,*N*‐dimethylformamide solution. They were then transferred to a dish containing medical gauze (MG). After drying, hydrophobic Cu_3_BiS_3_ NPs were embedded into PVDF‐HFP/MG (Abbreviated as PH) to form a multifunctional and biocompatible wound dressing (Abbreviated as PHC). Under isothermal conditions, the polarized charges on the surface of the PH are confined by internal compensation charges, leading to the absence of free electrons or holes. When introducing alternate NIR light (Figure 1B), the temperature variation induced by Cu_3_BiS_3_ NPs not only eradicated a portion of the bacteria but also changed the orientation of the internal dipoles of PVDF, resulting in the abundant release of confined charges. The released electrons and holes would react with water or oxygen molecules to produce ROS, resulting in effective bacterial inactivation. Moreover, under frequent stimulation by a movable wound, a continuous electrical field is generated owing to the piezoelectric performance of PVDF, which promotes cell migration and proliferation at the wound site. A series of in vitro and in vivo assessments demonstrated that PHC wound dressings effectively promoted the healing of nape wounds. Healing of wounds on the dorsum can be further accelerated using ultrasound. Furthermore, PHC wound dressings can be sterilized by alternating NIR light irradiation and reused to promote wound healing. The design of PHC wound dressings provides an opportunity to develop multifunctional bandages for wounds in movable parts based on piezo‐ and pyromaterials.

## 2 Results and Discussion

### 2.1 Preparation and Characterization of the PHC Wound Dressing

**FIGURE 2:**
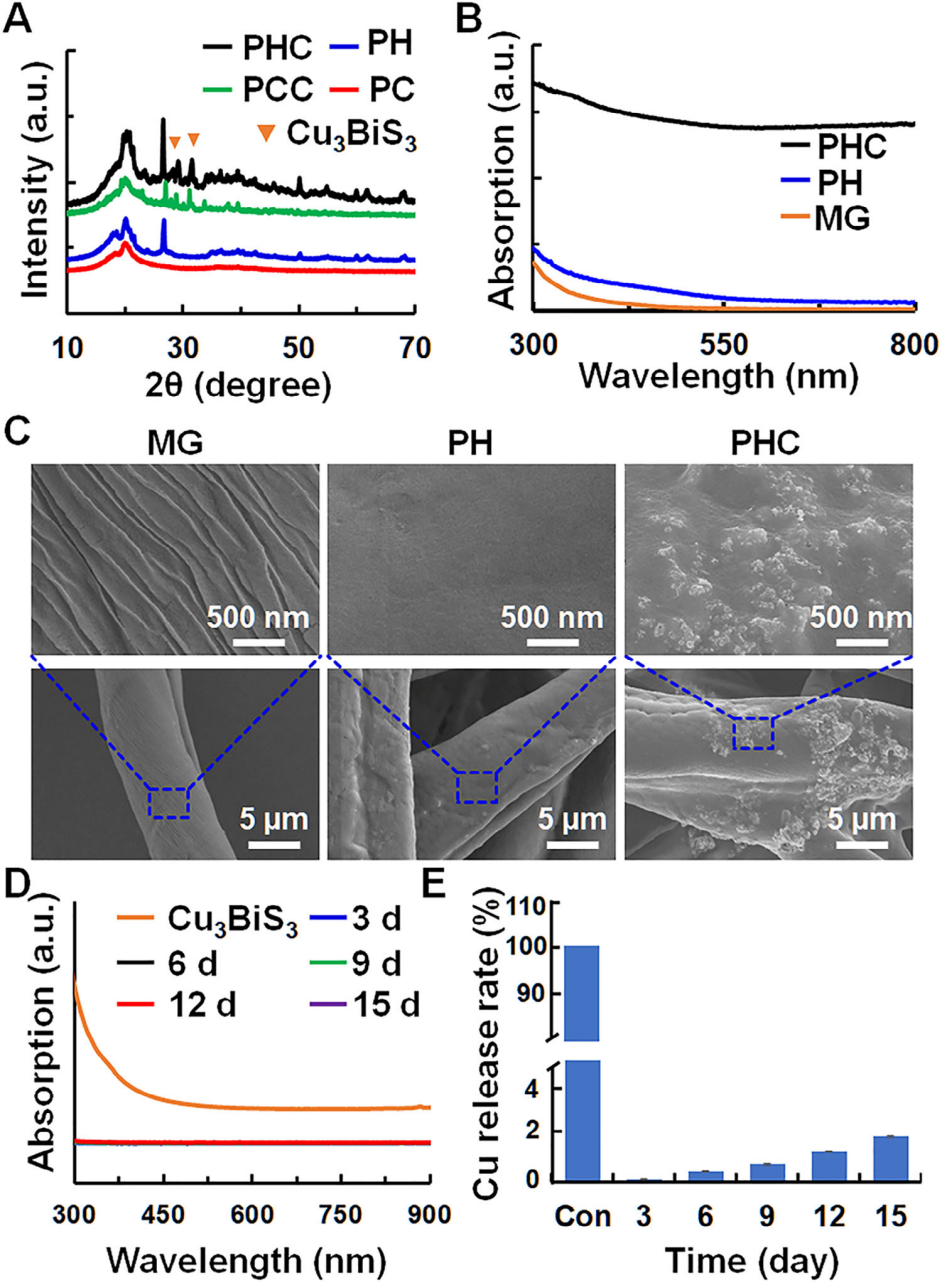
Physical and chemical properties of PHC wound dressing. (A) XRD patterns of PC, PH, PCC, and PHC wound dressings; (B and C) UV–vis diffuse reflectance spectra (B) and SEM images (C) of MG, PH and PHC wound dressings; (D and E) UV–vis spectra (D) and Cu ions concentration (E) of PBS solution containing PHC wound dressing at 0, 3, 6, 9, 12, 15 days.

Cu_3_BiS_3_ NPs were synthesized using a thermal injection method. The X‐ray diffraction (XRD) pattern (Figure S1, Supporting Information) demonstrated that all peaks could be attributed to the Cu_3_BiS_3_ (JCPDS: 43–1479) phase. The composition of the Cu_3_BiS_3_ NPs was studied by X‐ray photoelectron spectroscopy (XPS), and the results (Figure S2, Supporting Information) demonstrated the presence of Cu, Bi, and S elements. Transmission electron microscopy (TEM) imgae (Figure S3, Supporting Information) revealed that the Cu_3_BiS_3_ NPs were irregular in shape and approximately 50 nm in size. Finally, the ultraviolet‐visible (UV–vis) diffuse reflectance spectra (Figure S4, Supporting Information) showed that Cu_3_BiS_3_ has strong NIR absorption, supporting the idea that it can be excited by NIR light. To create a PHC wound dressing, a deposition process involving PH and Cu_3_BiS_3_ NPs on MG was employed. For comparison, polyvinylidene fluoride‐co‐chlorotrifluoroethylene (PC) lacks piezoelectric properties, was selected to form a PCC wound dressing using the same method. The XRD patterns (Figure 2A) displayed a diffraction peak located at 20.4°, corresponding to the (110) plane of β phase PVDF in PHC. Furthermore, peaks at 18.4, 19.9, and 26.7° were observed, aligning with (020), (110), and (021) planes of α phase PVDF, respectively. The peaks at 28.9° and 31.2° were identified as Cu_3_BiS_3_ NPs. The UV–vis diffuse reflectance spectra indicated significantly higher absorption in the NIR region for PHC and PCC than for PH and PC (Figure 2B and Figure S5, Supporting Information), owing to the incorporation of Cu_3_BiS_3_ NPs. This implied that the Cu_3_BiS_3_ NPs endowed the PHC with excellent photothermal properties.

Energy‐dispersive X‐ray spectroscopy (EDS) (Figure S6, Supporting Information) also demonstrated the successful synthesis of Cu_3_BiS_3_ in PHC wound dressings, and the mass fraction of Cu_3_BiS_3_ per 1 cm diameter of PHC was calculated to be 4.7%, which was similar to the content during fabrication. Optical microscopy and scanning electron microscopy (SEM) images (Figure 2C and Figure S7, Supporting Information) revealed a distinct difference between the MG and PHC. The surface of MG was rough, whereas the surface of PH was smooth, indicating that PH filled the gaps in MG. Meanwhile, the Cu_3_BiS_3_ NPs were embedded within the PVDF, thus preventing their detachment from the PHC. To test this hypothesis, PH and PHC were immersed in a PBS solution for 15 days, and the surface of PH was similar to that before immersion, as shown in the SEM image (Figure S8, Supporting Information), proving that PH was effectively attached to MG. In addition, the presence of Cu_3_BiS_3_ NPs in PBS was monitored by UV–vis spectroscopy and Cu‐based inductively coupled plasma‐optical emission spectrometry (ICP‐OES) every 3 days. As depicted in Figure 2D,E, there was almost no absorption of Cu_3_BiS_3_ NPs or Cu ions, indicating that the Cu_3_BiS_3_ NPs remained securely attached to the PHC throughout the immersion process. The mechanical properties of PHC were also tested as wound dressings. According to the force–displacement curves (Figure S9, Supporting Information), compared with the MG, the fracture strain of the PHC dressing significantly increased by 20%, and the fracture strength reached 48 KPa. In addition, the PHC showed good bendability and foldability (Figure S10, Supporting Information), which can better fit the wound in the movable part. Furthermore, the swelling capacity of the PHC wound dressing reached 22 g water/g watersorb.

### 2.2 Piezoelectric Property and ROS Generation Ability of PHC Wound Dressings

**FIGURE 3:**
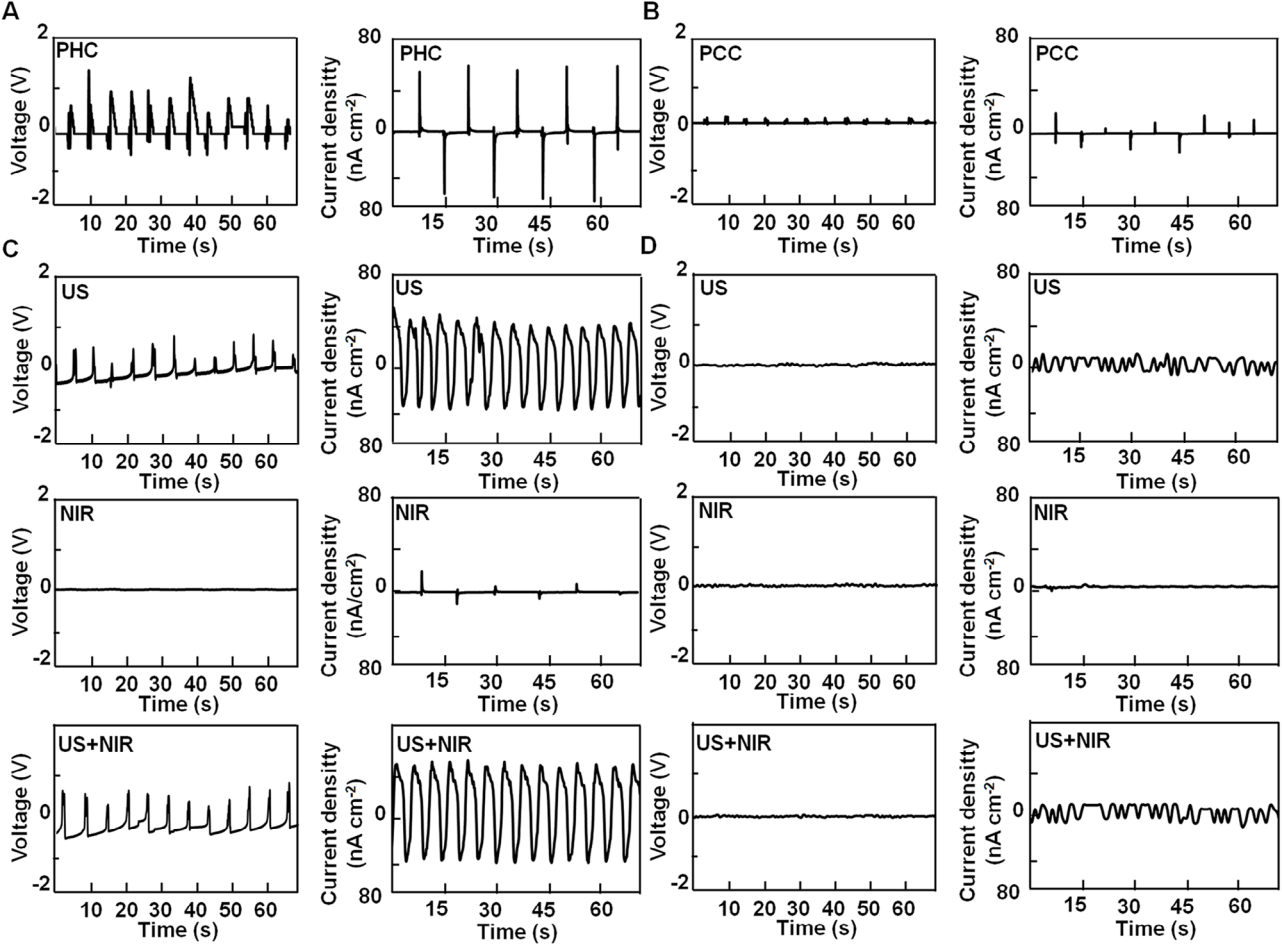
Piezoelectric voltage and current density generated by PHC (A and C) and PCC (B and D) wound dressings (patch size = 2 × 2 cm^−2^, bending radius = 5 mm) under mechanical bending (A and B), as well as ultrasound (US: 1 MHz, 0.5 W cm^−2^, 50% duty cycle), 808 nm laser irradiation (NIR: 0.5 W cm^−2^), and US+NIR (C and D).

To prove the electric field‐producing ability of the PHC wound dressing from self‐motion, the piezoelectric voltage and current density were measured in response to the mechanical deformation of the PHC wound dressing. As shown in Figure 3A,B, the voltage and current density generated on the PHC wound dressing surface upon bending with a 5 mm bending radius were about 1.5 V and 60 nA cm^−2^, respectively, while PCC wound dressing could produce much weaker voltage and current. We also tested the voltage and current generated by the PHC and PCC wound dressings on the nape and back of the mouse. No current or voltage was detected on either the PHC or PCC wound dressings when they were attached to the backs of the mice (Figure S11, Supporting Information). However, when they were attached to the nape, a voltage of ∼0.85 V could be produced in PHC wound dressing while not PCC (Figure S12, Supporting Information), indicating that self‐motion of mice can be used for activating PHC piezo wound dressing. We also measured the current and voltage generated by the PHC and PCC wound dressings with or without NIR light (or ultrasound) stimulation. For the PHC wound dressing (Figure 3C), a current or voltage was produced when ultrasound was used. As shown in Figure 3D, the PCC wound dressing did not produce any current or voltage when stimulated by NIR light, ultrasound, or both. Therefore, ultrasound can also be used to activate PHC piezo wound dressings.

**FIGURE 4:**
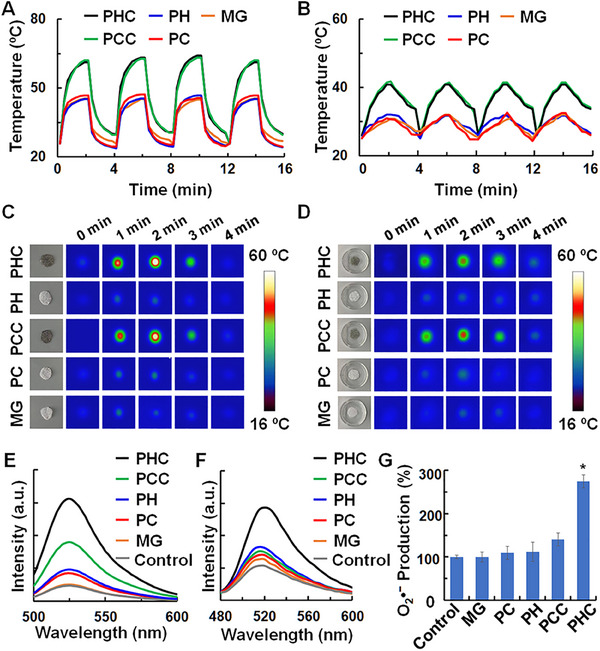
Photothermal and pyro‐mediated ROS generation ability of PHC wound dressing. (A–D) Photothermal curves (A and B) and infrared thermal images (C and D) of MG, PC, PH, PCC, and PHC (1 cm in diameter) wound dressings (A and C) and their aqueous solution (B and D) with alternated 808 nm laser irradiation (0.5 W cm^−2^, 2 min, 4 cycles); (E–G) DCF and APF fluorescence spectra, as well as O_2_•^−^ kits for total ROS (E), OH• (F) and O_2_•^−^ (G) detection, in which the detection agents were incubated with PBS, MG, PC, PCC, PH and PHC (1 cm in diameter) with alternated 808 nm laser irradiation (0.5 W cm^−2^, 2 min, 4 cycles).

Leveraging the pyroelectric properties of PH and photothermal attributes of Cu_3_BiS_3_ NPs, we employed alternating NIR light irradiation to induce temperature changes in the PHC wound dressing, thereby facilitating ROS production. Consequently, we investigated the photothermal performances of the PHC. As shown in Figure 4A, when subjected to 808 nm laser irradiation (0.5 W cm^−2^, 2 min, 4 circles), the temperature of the PHC wound dressing rose from 24 to 60°C, a substantial 20°C higher than that of PH alone. Even when the PHC dressing was immersed in an aqueous solution, the temperature increased from 24 to 40°C, surpassing that of PH by 10°C (Figure 4B). Similar temperature changes were observed for the PCC and PC wound dressings. Remarkably, after irradiation with NIR light for four cycles, the temperature variation remained stable. Furthermore, infrared thermal images of the PHC dressing (Figure 4C) and aqueous solution containing PHC (Figure 4D) provide additional evidence supporting the photothermal performance of the PHC wound dressings under alternating NIR light irradiation. Subsequently, the overall ROS generation ability was assessed using the 2′,7′‐dichlorodihydro‐fluorescein diacetate (H_2_DCFDA) agent, which could be oxidized into 2′,7′‐dichlorodihydrofluorescein (DCF) with strong fluorescence. As illustrated in Figure 4E, PHC exhibited a significantly higher fluorescence intensity of DCF than PH with alternate 808 nm laser irradiation (0.5 W cm^−2^, 2 min, 4 cycles), whereas PCC and PC dressings failed to generate any fluorescence. Without alternated NIR laser irradiation, no DCF fluorescence was induced (Figure S13, Supporting Information), indicating that PHC wound dressing's ROS generation efficiency can be attributed to the integration of photothermal Cu_3_BiS_3_ NPs and pyroelectric PH. To delve deeper into the specific type of ROS generated by the PHC wound dressing, we employed 3′‐(p‐aminophenyl) fluorescein (APF) and superoxide anion detection kits to evaluate hydroxyl radicals (OH•) and superoxide (O_2_•^−^), respectively. As depicted in Figure 4F,G, with 808 nm laser irradiation (0.5 W cm^−2^, 2 min, 4 cycles), the PHC wound dressing exhibited a greater production of OH• and O_2_•^−^ compared to PH, whereas PCC and PC dressings showed negligible generation. In addition, without NIR light irradiation, none of the dressings produced OH• and O_2_•^−^ (Figure S14, Supporting Information). These results collectively validate the successful fabrication of a PHC wound dressing with exceptional photothermal and pyro‐mediated ROS generation capabilities.

### 2.3 Antibacterial Activity of PHC Wound Dressings

**FIGURE 5:**
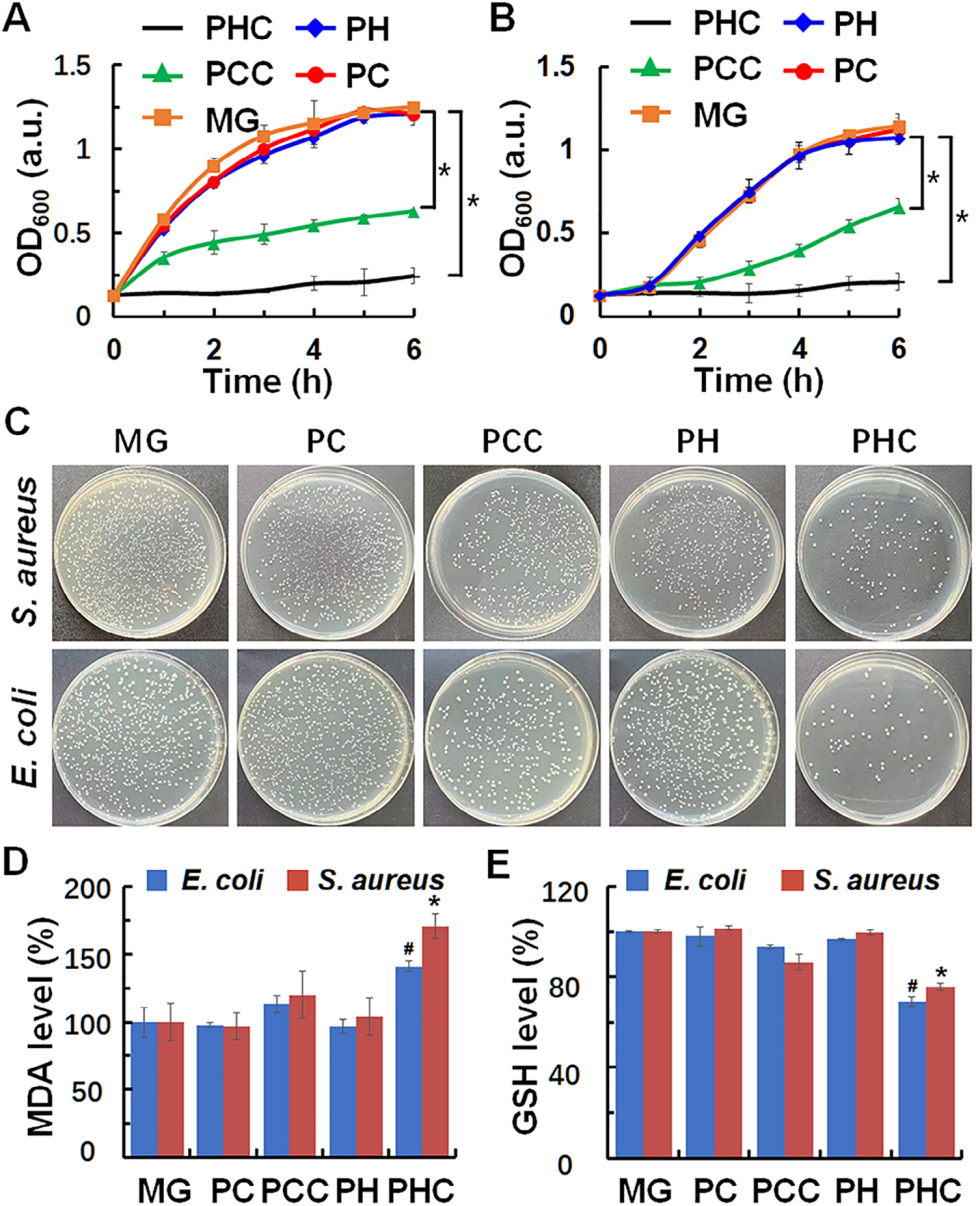
Antibacterial capabilities of PHC wound dressing. (A and B) The bacterial growth curves of *S. aureus* (A) and *E. coli* (B); *n* = 3, *p < 0.05; (C) The optical images of bacterial colonies of *S. aureus* and *E. coli*; (D and E) MDA (D) and GSH (E) levels in *S. aureus* and *E. coli; n* = 3, #*p* < 0.05, *p < 0.05. For (A–E), *S. aureus*, and *E. coli* were treated with MG, PC, PCC, PH, and PHC (1 cm in diameter) with alternated 808 nm laser irradiation (0.5 W cm^−2^, 2 min, 4 cycles).

Bacterial infections remain a major obstacle to skin trauma and tissue reconstruction. Inspired by the heat and ROS generation abilities of PHC dressings under NIR light irradiation, we evaluated their antibacterial efficacy against gram‐positive *S. aureus* and gram‐negative *E. coli*. As depicted in the bacterial growth curves of both bacteria incubated with PHC, PCC, PH, or PC wound dressings (Figure 5A,B), PHC effectively inhibited bacterial growth, surpassing the performance of PCC wound dressings upon exposure to alternated 808 nm laser irradiation (0.5 W cm^−2^, 2 min, 4 cycles). Conversely, no antibacterial activity was observed for the other wound dressings. In the absence of irradiation (Figure S15, Supporting Information), none of the wound dressings exhibited inherent antibacterial capacity. The results of bacterial colony (Figure 5C and Figure S16, Supporting Information) and the corresponding statistical analysis (Figure S17, Supporting Information) showed that the bacteriostatic rates of the PHC wound dressing against *E. coli* and *S. aureus* were 82.12% and 96.74%, respectively. ROS and heat damage the bacterial membrane, leading to lipid peroxidation and significantly increased cellular ROS, thereby depleting the bacterial cellular intracellular glutathione (GSH) [27]. To further confirm the antibacterial potential of the PHC wound dressing, we assessed bacterial lipid peroxidation and GSH levels using malondialdehyde (MDA) production and 5,5'‐dithiobis‐(2‐nitrobenzoic acid) assay, respectively. As shown in Figure 5D,E, the PHC wound dressing not only induced higher MDA levels, damaging bacterial cell membranes, but also consumed more GSH, thus reducing the antioxidant capacity of bacteria upon exposure to alternating 808 nm laser irradiation (0.5 W cm^−2^, 2 min, 4 cycles). Conversely, without irradiation, the MDA and GSH levels remained unchanged (Figure S18, Supporting Information). Collectively, these results proved that the PHC wound dressing effectively eradicated both Gram‐negative and Gram‐positive bacteria owing to its excellent ROS and heat generation abilities.

### 2.4 Cell Migration and Proliferation Induced by the PHC Wound Dressings

**FIGURE 6:**
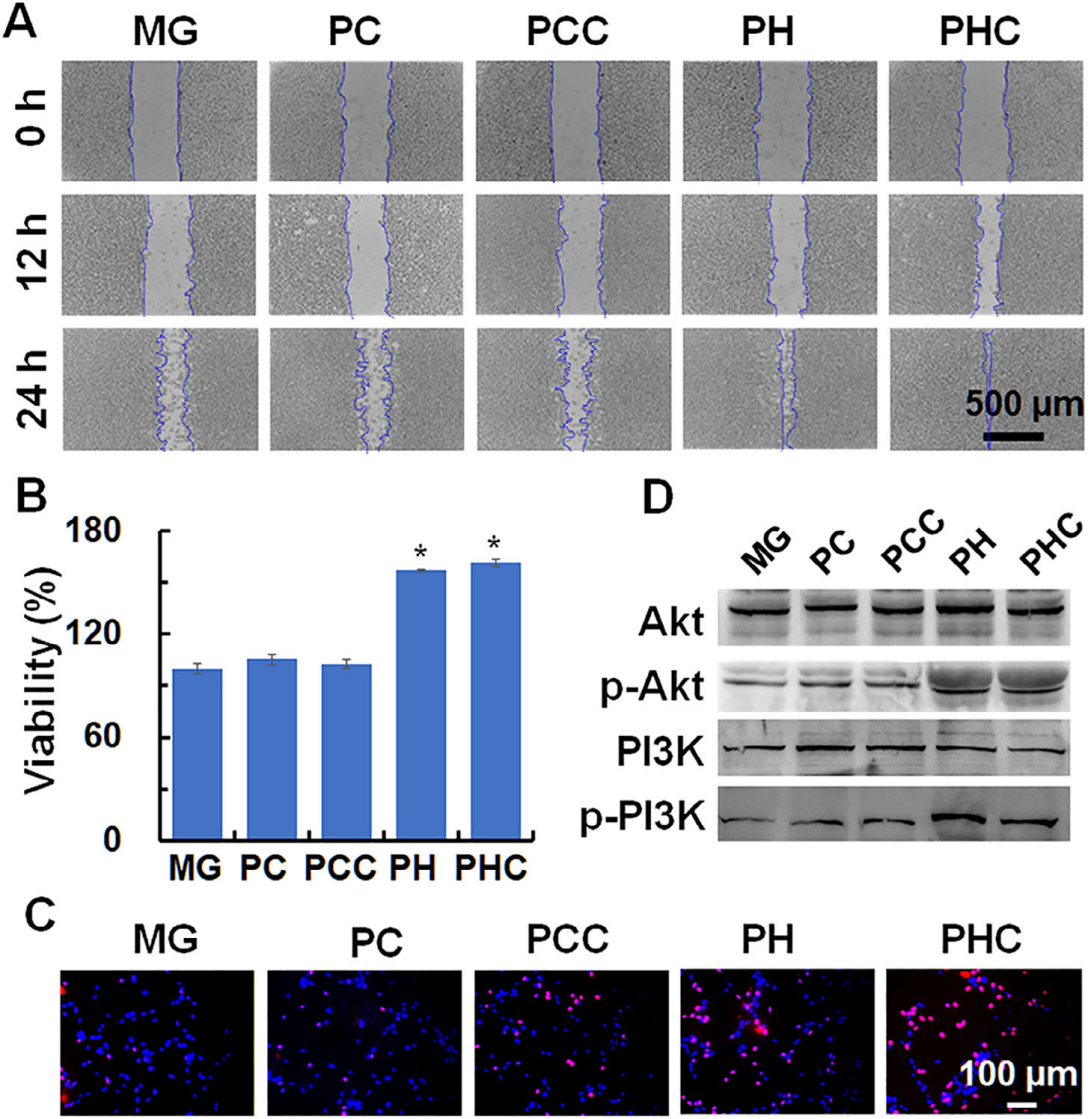
Cell migration and proliferation induced by PHC wound dressing. (A) Optical images of scratch assay performed on NIH‐3T3 cells treated with MG, PC, PCC, PH, PHC (2 cm in diameter in 12‐well plate) at 0, 12, and 24 h; (B) Viabilities of NIH‐3T3 cells treated with MG, PC, PCC, PH, and PHC (1 cm in diameter in 24‐well plate) wound dressing for 24 h; *n* = 3, *p < 0.05, compared with MG+US group; (C) Fluorescence images of EdU (red) and Hoechst 33342 (blue) stained NIH‐3T3 cells treated with MG, PC, PCC, PH, and PHC (1 cm in diameter in 24‐well plate); (D) Akt, pAkt, PI3K, pPI3K expressions of NIH‐3T3 cells treated with MG, PC, PCC, PH, and PHC (2 cm in diameter in 12‐well plate) wound dressing. For (A–D), all the cells were irradiated with ultrasound (1 MHz, 0.5 W cm^−2^, 50% duty cycle, 10 min).

Electric fields generated by exogenous electrical stimulation effectively promote cell migration and proliferation [28]. Capitalizing on the piezoelectric properties of PHC, we introduced ultrasound to simulate stress generation in the cells. To evaluate the effect of PHC wound dressing on cell migration and proliferation, mouse fibroblasts (NIH‐3T3 cells) were co‐cultured with MG, PC, PCC, PH, or PHC for 24 h with or without ultrasound stimulation. The scratch wound healing assay (Figure 6A and Figure S19, Supporting Information) revealed that cells treated with PHC and PH displayed higher migrating rates compared to those treated with PCC and PC when subjected to ultrasound stimulation, cell mobility in PH and PHC group was 33.98 ± 3.02% and 38.07 ± 1.33% higher than that in MG group, respectively. Without ultrasound stimulation (Figures S20 and S21, Supporting Information), no substantial difference was observed in the proliferative ability of MG‐, PC‐, PCC‐, PH‐, and PHC‐treated cells, proving that PHC and PH wound dressings promote cell migration owing to their piezoelectric properties. Next, cell proliferation ability was accessed by 3‐(4,5‐dimethylthiazol‐2yl)‐2,5‐diphenyl‐2H‐tetra‐zolium bromide (MTT) and 5‐ethyl‐2'‐deoxyuridine (EdU) assays. The MTT assay demonstrated that cell viabilities were 161.49 ± 2.06% and 157.20 ± 0.88% in NIH‐3T3 cells treated with PHC and PH wound dressing with ultrasound stimulation, while cell viabilities for PCC and PC‐treated cells were only 102.77 ± 2.60% and 105.42 ± 2.87%, respectively (Figure 6B), while no variations were found without ultrasound stimulation (Figure S22, Supporting Information). The EdU assay (Figure 6C and Figure S23, Supporting Information) further supported these results, showing that the proliferation of NIH‐3T3 cells was significantly increased by the PHC and PH wound dressings after ultrasound stimulation. No proliferation was observed in the absence of US stimulation. Previous studies indicated that electric fields generated by exogenous electrical stimulation can accelerate the migration and proliferation of key cells through enhanced phosphorylation of protein kinase B (Akt) and phosphoinositide 3‐kinase (PI3K) [26]. As depicted in Figure 6D and Figure S24, Supporting Information, with ultrasound simulation, the levels of phosphorylated PI3K and AKT significantly improved in cells treated with PH and PHC wound dressings. Taken together, ultrasound stimulation with a PHC wound dressing effectively promoted cell migration and proliferation through the phosphorylation of Akt and PI3K.

### 2.5 Biocompatibility and Hemostasis of PHC Wound Dressings

Biocompatibility is a critical requirement for wound dressings because of their direct contact with wounds. First, the hemocompatibility of the PHC wound dressings was analyzed using an in vitro hemolysis test. As shown in Figure S25, Supporting Information, after treatment with the PHC wound dressing, the hemolysis rate was only 2.86%, indicating that the dressing exhibited good blood compatibility. We also assessed the biocompatibility of wound dressings throughout the immersion process. As shown in Figure S26, Supporting Information, the extracts of the MG, PC, PCC, PH, and PHC wound dressings after soaking in PBS for 15 days showed no toxicity to NIH‐3T3 cells. To evaluate the skin sensitization of the PHC wound dressing, 2,4‐ dinitro‐chlorobenzene (positive control) and a PHC bandage were applied to the skin of the mice for 72 h. The skin reaction intensity in each group was graded using a sensitization score [29]. As shown in Table 1, erythema, edema, or other allergic symptoms were only observed in 2,4‐dinitro‐chlorobenzene (C_6_H_3_CLN_2_O_4_)‐treated skin but not in PHC‐treated skin, indicating good biocompatibility of the PHC wound dressing. Additionally, the acute systemic toxicity of PHC wound dressings was evaluated. There were no changes in body weight or other toxic symptoms between mice treated with saline and the PHC extract within 3 days (Table 2). This indicates that the PHC wound dressing is a relatively safe and non‐toxic option for wound treatment, providing a promising alternative for clinical treatment. The hemostatic ability of the PHC wound dressing was determined in a mouse model with a severed tail. MG or PHC wound dressings were applied immediately after tail incision. The amount of blood loss was quantitatively analyzed by comparing the weight of the filter paper before and after the incision. The results (Figure S27, Supporting Information) indicated that the PHC wound dressing had a hemostatic ability similar to that of the MG.

**TABLE 1:** Allergenic effects of PHC wound dressings on mice skin (*n* = 5).

	Total score					
Group	1 h	24 h	48 h	72 h	The rate of sensitization (100%)	Intensity of allergic reaction
PBS	0	0	0	0	0	No
PHC extract	0	0	0	0	0	No
C_6_H_3_ClN_2_O_4_	6	22	32	26	100	Yes

**TABLE 2:** The results of acute toxicity test for PHC wound dressings.

Group	Animal number	Observe the number of days	Weight	Mortality
Control	5	3	+1.65 g	0
PHC soak liquid	5	3	+1.71 g	0

### 2.6 In Vivo Wound Healing Performance of the PHC Wound Dressings

Leveraging the exceptional capacities of antibacterial action as well as cell proliferation and migration promotion, we conducted in vivo experiments to assess the wound healing ability of PHC wound dressings in both full‐depth bacteria‐infected wounds in the movable (nape) and inactive (back) parts. Prior to the in vivo experiment, PHC bandages (Figure S28, Supporting Information) were fabricated on the base of the MG and 3 M adhesives. The ability to maintain breathability is essential for wound dressings, as it allows carbon dioxide transfer, oxygen entry, and excess exudate evaporation, all of which are conducive to normal metabolic function. To ensure proper permeability, the water vapor transmittance of PHC and PCC bandages was measured to be 0.22 and 0.23 g cm^−2^ per day, respectively, falling within the optimal range, confirming the well‐permeable properties. The fabricated PHC bandage exhibited good adhesion and bendability at the bent knuckles, as shown in Figure S29, Supporting Information.

**FIGURE 7:**
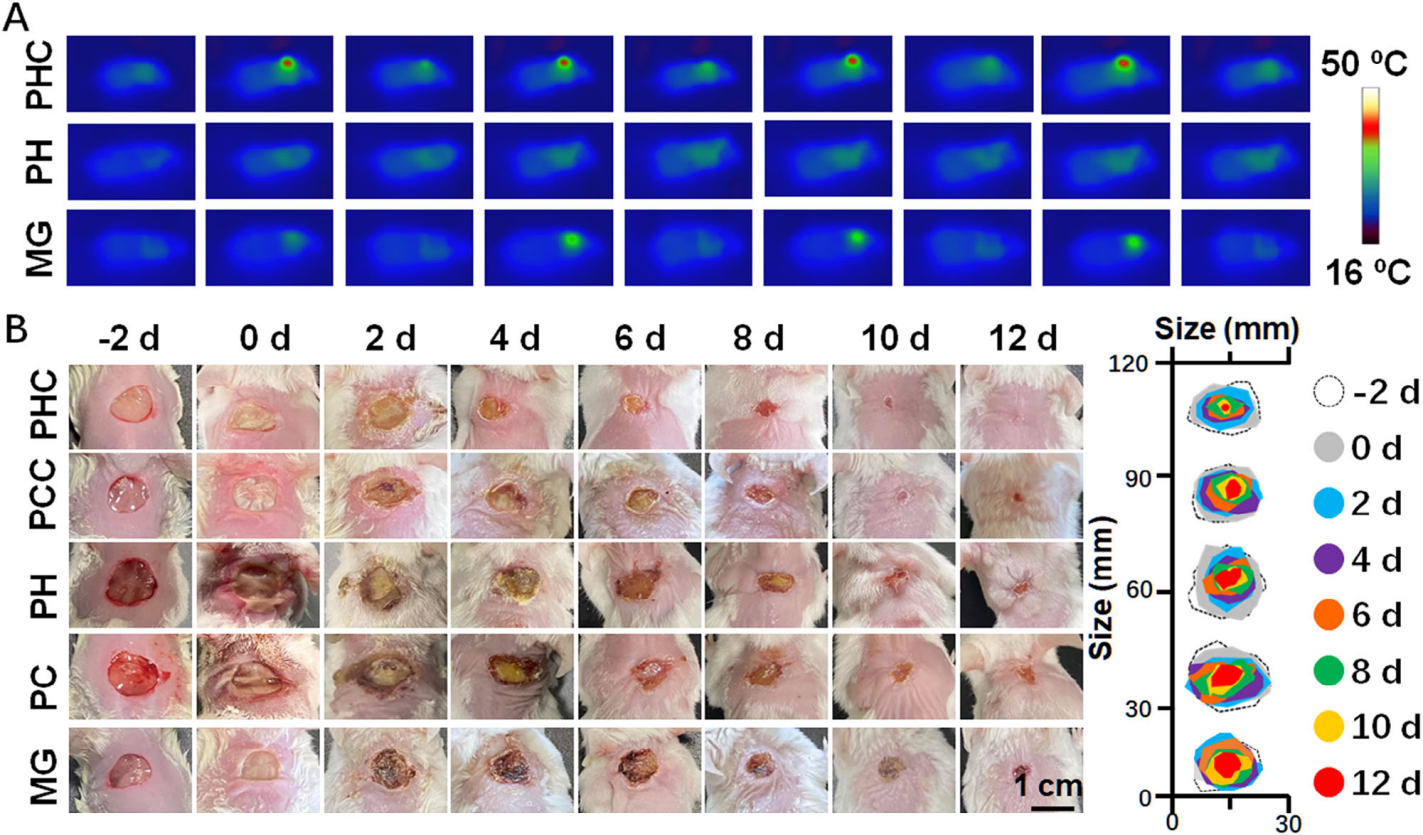
In vivo wound healing effect of PHC bandage. (A) In vivo thermal images of MG, PH, and PHC wound dressings treated nape wound with alternated 808 nm laser irradiation (0.5 W cm^−2^, 2 min, 4 cycles); (B) The representative images and the magnified simulation of the wound size of MG, PC, PCC, PH, and PHC treated nape wounds on days −2, 0, 2, 4, 6, 8, 10, and 12 with alternated 808 nm laser irradiation (0.5 W cm^−2^, 2 min, 4 cycles).

The wound‐healing capability was then investigated. For wounds on the nape, alternating NIR light irradiation was employed to activate the pyroelectric performance for bacterial inactivation. Thermal imaging visually depicts the photothermal effects of the wound dressings. As shown in Figure 7A and Figure S30, Supporting Information, the temperature of the wound increased to 46°C with alternated 808 nm laser irradiation (0.5 W cm^−2^, 2 min, 4 cycles) due to the addition of Cu_3_BiS_3_ NPs in PHC and PCC bandages, while the temperature is 34, 35 and 40°C in PH, PC, and MG treated wounds, respectively. After bacterial inactivation, the motion of the nape stimulated the piezoelectric properties of the PHC. As shown in Figure S31, Supporting Information, at 12 days post‐application of the bandage, the wound closure rate of the PHC‐treated wound was 97.94 ± 1.31%, higher than that of the PH wound dressing due to the stronger antibacterial ability of PHC. Moreover, the closure rate of the PH‐treated wound is higher than that of both PC and MG whether irradiated with or without alternated 808 nm laser (0.5 W cm^−2^, 2 min. 4 cycles), proving that the piezoelectric performance induced by the nape motion can effectively promote wound healing. Representative images of the mice and wounds (Figure 7B and Figure S32, Supporting Information) further confirmed that PHC could accelerate the healing process of the wound in the movable parts.

**FIGURE 8:**
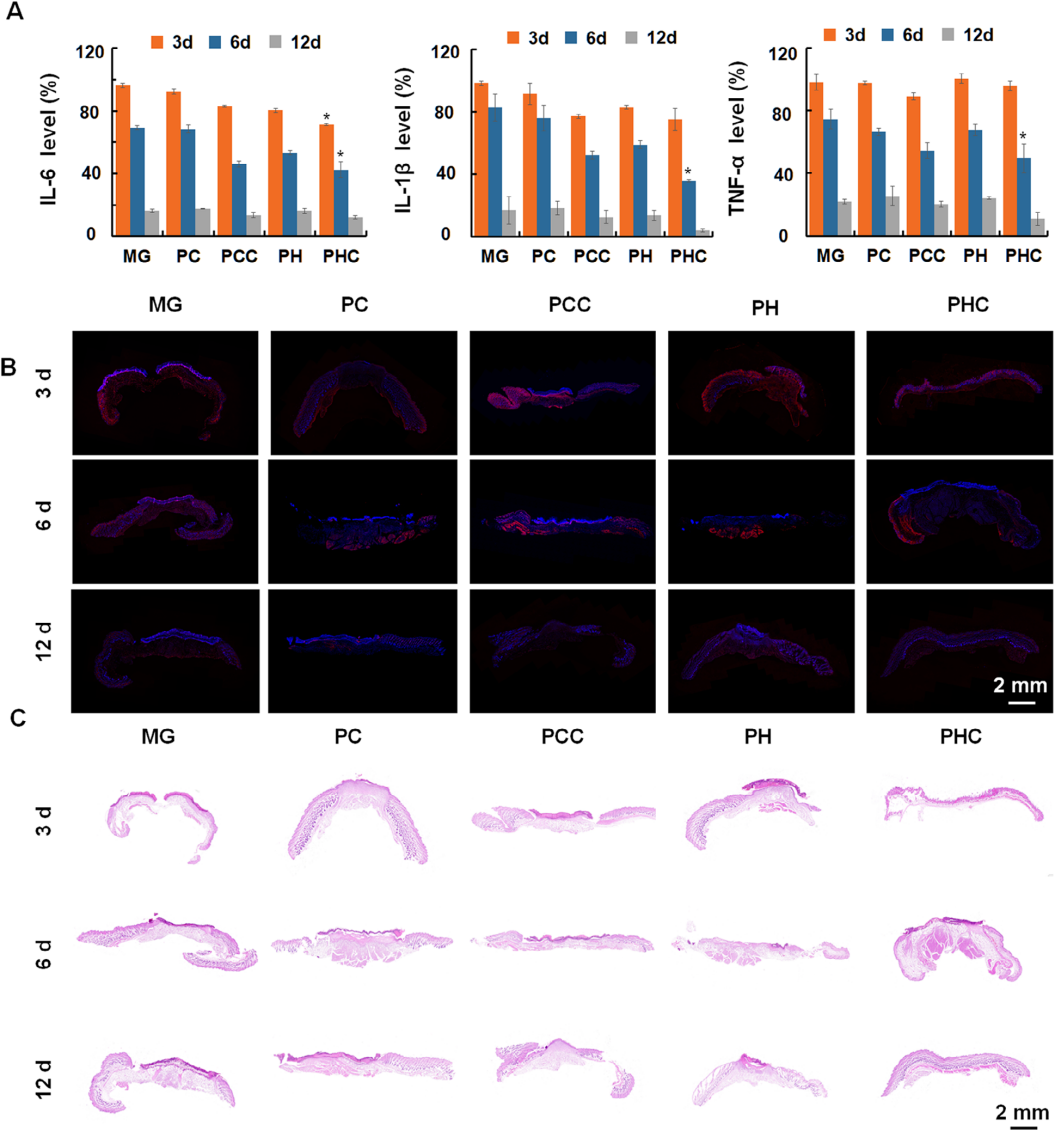
Mechanism study of wound healing process. IL‐6, IL‐1β, and TNF‐α levels accessed by ELISA (A), immunofluorescence staining images of IL‐6 (B) in wound tissues, as well as H&E staining images (C) of wound tissues at 3, 6, and 12 days after being treated with MG, PC, PH, PCC and PHC with alternated 808 nm laser irradiation (0.5 W cm^−2^, 2 min, 4 cycles); *n* = 3, *p < 0.05, compared with MG group; For immunofluorescence staining images, the blue and red fluorescence represents nuclei labeled with DAPI dye and inflammatory cytokines labeled with fluorescent antibodies, respectively.

After skin damage, inflammatory factors can accumulate in damaged wounds, and their levels are reduced in the next stage of the healing process [30]. Therefore, the inflammatory cytokines including IL‐6, IL‐1β, and TNF‐α in wound tissues were detected by ELISA and immunofluorescence staining. Owing to the photothermal treatment of wound dressings in the PHC and PCC groups, most bacteria were killed, and inflammation was reduced. As shown in Figure 8A,B and Figure S33–S35, Supporting Information, the PHC and PCC groups secreted lower levels of inflammatory cytokines than the other groups 3 days post‐antibacterial treatment. 6 days after treatment, the levels of the inflammatory cytokines in each group were further reduced. PHC‐treated mice showed the lowest levels because the wound healing process developed faster in the PHC group than in the other groups. On the 12th day after treatment, the wounds in each group had almost completely healed, and almost no inflammatory cytokines were expressed in the tissues. Furthermore, histological analysis of wound tissues was performed using hematoxylin and eosin (H&E) staining at 3, 6, and 12 days after antibacterial treatment. As shown in Figure 8C, the wounds in all groups were in the initial stage of healing on day 3 with no significant differences. On the 6th day, wound healing began and the healing rate and epidermal layer growth in the PHC wound dressing group were higher than those in the other groups. On the 12th day, wound healing entered the late stage, the epithelium was intact, and wound healing was complete.

To further substantiate the hypothesis that the stress produced by the frequent activity of movable wounds could trigger piezoelectric materials to accelerate wound healing, a PHC bandage was applied to the wounds on the backs of the mice. Thermal images (Figure S36, Supporting Information) indicated that the temperature variation at the wound site induced by PHC was similar to that on the nape wound, implying that PHC exhibited similar antibacterial activity in both wounds. Figure S37, Supporting Information shows the wound closure rate and representative images of the wound, where the PHC bandage showed a slightly higher wound closure rate than the PCC bandage with or without NIR light irradiation, indicating that animal motion had a marginal effect on the activation of the piezoelectric performance. However, when ultrasound was used to stimulate PHC instead of nape motion, the PHC bandage displayed a much higher wound closure rate than the PCC bandage because of the stimulation of the piezoelectric performance of PH. As a result, the multifunctional PHC wound dressing could effectively promote the healing process of wounds in movable parts owing to frequent motion.

### 2.7 Reusability of PHC Wound Dressings

**FIGURE 9:**
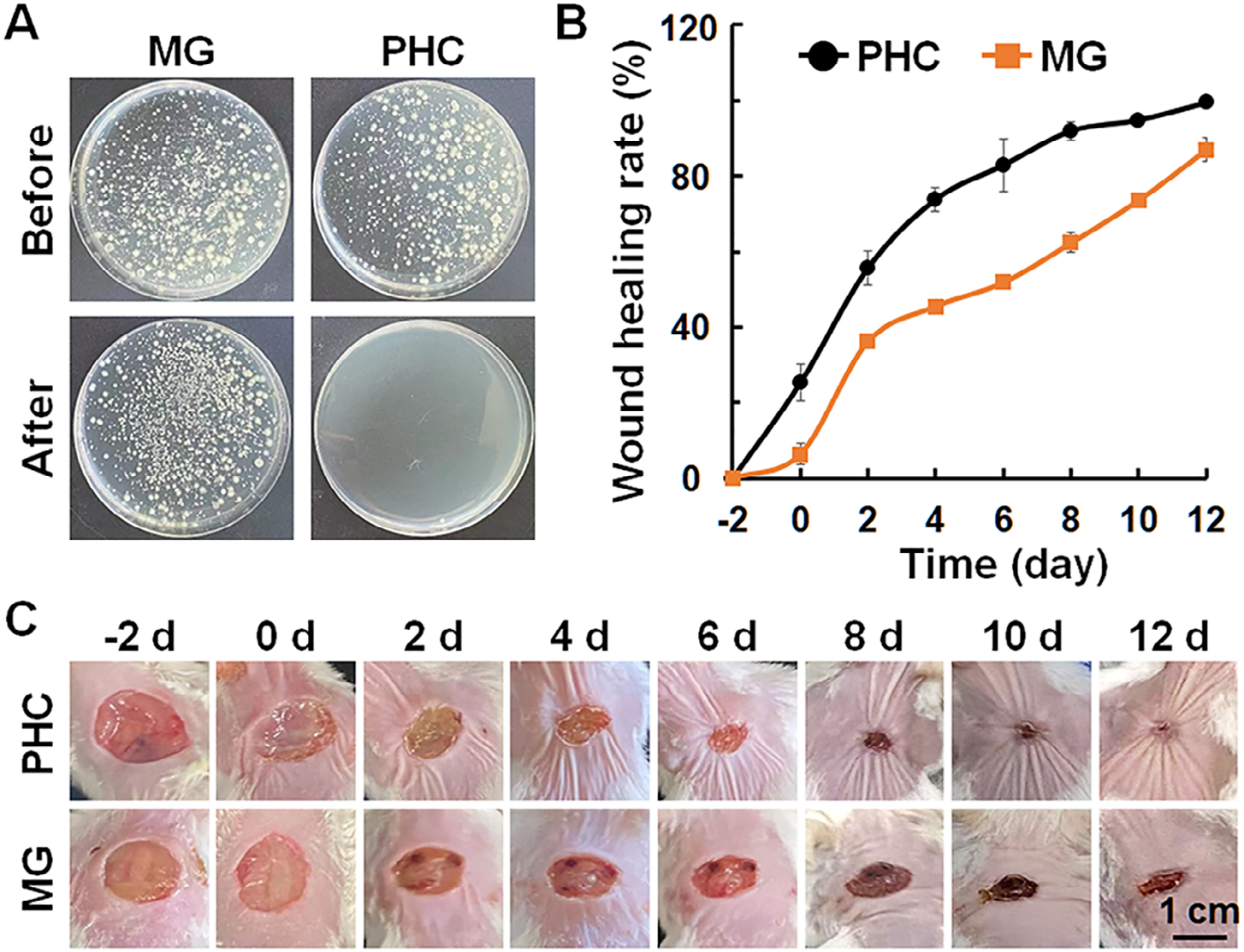
The reusability of PHC wound dressing. (A) Optical images of bacterial colonies from the reusable MG and PHC wound dressings before and after alternated 808 nm laser irradiation (1 W cm^−2^, 2 min, 6 cycles); (B and C) Wound closure rates (B) and the representative photos of the wound (C) treated with MG and sterilized PHC bandage with alternated 808 nm laser irradiation (0.5 W cm^−2^, 2 min, 4 cycles).

Reusability is another key factor in wound dressings that reduces costs and facilitates further applications. Owing to the excellent antibacterial activity of the PHC wound dressing with NIR light irradiation, we speculated that the PHC bandage could be self‐cleaning. After wound healing, the PHC wound dressing was recycled and washed three times with PBS. An alternating NIR light was used to sterilize PHC wound dressings. As shown in Figure 9A, no bacteria were observed after NIR light sterilization. Most importantly, the sterilized PHC wound dressing exhibited similar wound healing performance when used to fabricate a bandage for nape wound healing (Figure 9B,C). Therefore, PHC can be used as wound dressings with excellent reusability.

## 3 Conclusion

In conclusion, we developed a multifunctional, reusable PHC wound dressing that expedites the healing of nape wounds. This pioneering dressing harnesses the combined power of Cu_3_BiS_3_ NPs' photothermal performance and PVDF's pyroelectric property, allowing for abundant heat generation and ROS production upon exposure to alternating NIR light, effectively inactivating bacteria. Additionally, the piezoelectric attributes of PVDF facilitate cell migration and proliferation, synergistically promoting wound healing through the frequent movement of the nape. Furthermore, PHC wound dressings can be easily sterilized and rendered reusable by alternating NIR irradiation, making them a sustainable solution for wound healing. This breakthrough study not only highlights the potential of multifunctional bandages for movable parts but also opens new avenues in the field of wound care, spearheading progress in the integration of piezo‐ and pyro‐materials.

## 4 Experimental Section

Experimental details are provided in the Supporting Information.

## Author Contributions

**Liqi Wei**: investigation, conceptualization, methodology, writing – original draft. **Xin Liu**: methodology, data curation. **Yuanqiang Li**: formal analysis, methodology. **Yu Han**: formal analysis, methodology. **Yiping Ren**: formal analysis, methodology. **Tianshu Zou**: formal analysis, methodology. **Pengcheng Yu**: data curation, software. **Yining Chen**: data curation, software. **Biao Zhang**: data curation, software. **Zixuan Wang**: data curation, software. **Jingyi Jiang**: data curation, software. **Yumi Kim**: data curation, software. **Rui Chen**: supervision, conceptualization, resources, project administration, funding acquisition, writing – review & editing. **Yan Cheng**: supervision, conceptualization, resources, project administration, funding acquisition, writing – review & editing. **Hongxia Ma**: supervision, conceptualization, resources, project administration, funding acquisition, writing – review & editing.

## Ethical Statement

Female ICR mice were purchased from the Experimental Animal Center of Jilin Agricultural University, and all animal experiments were conducted at the Animal Center of Jilin Agricultural University (SYXK (Ji) 2023–0021).

## Conflicts of Interest

The authors declare no conflicts of interest.

## Supporting information



Supporting Information

## Data Availability

All data needed to support the findings of this study are present in the paper and/or in the Supporting Information. Additional data related to this paper are available from the corresponding authors.
