# Supplementary material for: Multifunctional PHC Bandage for Accelerated Wound Healing in Movable Parts
Source: Exploration (Beijing). 2025 Mar 6;5(3):20230176. doi: 10.1002/EXP.20230176 (PMC12199424; doi:10.1002/EXP.20230176)
Supplement: Supplementary file 1 — Supporting Information [file EXP2-5-20230176-s001.docx]

Supporting Information

**Multifunctional PHC Bandage for Accelerated Wound Healing**

**in Movable Parts**

*Liqi Wei, ^1,#^ Xin Liu ^1,#^ Yuanqiang Li,^1^ Yu Han,^1^ Yiping Ren,^1^ Tianshu Zou,^1^ Pengcheng Yu,^2^ Yining Chen,^1^ Biao Zhang,^1^ Zixuan Wang,^1^ Jingyi Jiang,^1^ Yumi Kim,^3^ Rui Chen,* ^2^ Yan Cheng,* ^1^ and Hongxia Ma* ^1^*

^1^ Engineering Research Center of the Chinese Ministry of Education for Bioreactor and Pharmaceutical Development, College of Life Science, Jilin Agricultural University, Changchun, China

^2^ Jilin Provincial Key Laboratory of Human Health Status Identification and Function Enhancement, School of Materials Science and Engineering, Changchun University

^3^ Department of Biomedical Engineering, Ulsan National Institute of Science and Technology (UNIST), Ulsan, Republic of Korea

*^#^* These authors contributed equally to this work.

Experimental Section

1 Synthesis of Cu_3_BiS_3_ NPs

Typically, 0.363 g of bismuth nitrate pentahydrate and 0.589 g of copper acetylacetone were uniformly dispersed in 12 mL of olamine and stirred for 1 h at 140 °C. Subsequently, the solution was subsequently heated to 220 °C and maintained for 10 min. Then, 2.5 mL of oleyamine containing thioacetamide (0.228 g) was added dropwise under vigorous stirring. After stirring for 30 min at 220 °C, the solution was cooled to room temperature and washed with the ethanol several times with ethanol. Finally, the obtained Cu_3_BiS_3_ NPs were dispersed in 10 mL of chloroform for further use.

2 Synthesis of PHC wound dressing

Firstly, 10 mL of dimethylformamide containing 1 g PVDF-HFP (or PVDF-CTFE) and 2 mL of the above Cu_3_BiS_3_ NPs was mixed and stirred to achieve a uniform dispersion. Then the above solution was poured into a glass container containing MG, and dried at 100 ºC to obtain PHC (or PCC) wound dressings. PH and PC wound dressings were prepared using the same protocol without the addition of Cu_3_BiS_3_ NPs.

3 Physicochemical characterizations

TEM images were obtained using transmission electron microscopy (JEM-2100F, JEOL, Japan). SEM images and EDS energy spectra were oobtained using Scanning Electron Microscopy (S-4800-II, HITACHI, Japan). XRD patterns were recorded using an X-ray diffractometer (D2, Bruker, Germany). UV-Vis spectra were recorded on a UV-Vis spectrophotometer (UV-3600i Plus, Shimadzu, Japan). The current and voltage generated by the wound dressing (2×2 cm^2^) were measured using a Keithley 6487 picoammeter (Keithley Instruments Inc., Cleveland, OH, USA) and an electrometer/high resistance meter (Keithley 6517, Keithley Instruments Inc.), respectively.

4 Mechanical property

The tensile properties of PHC and MG were measured by universal testing machine. The bending and folding ability of PHC dressing were tested and photographed. The monitoring of the PHC wound dressing’s weight increase in distilled water was conducted at room temperature for 48 h. The samples were removed from the water and their surfaces were carefully wiped with dry filter paper for weight purposes. The swelling capacity (SW) was then determined according to the following equation.

SW(%)= (Ws–Wd)/W_d_×100%

Where Ws and W_d_ are the weight of the swollen and dried dressings respectively.

5 Photothermal performance of PHC wound dressing

MG, PC, PCC, PH, and PHC wound dressings (1 cm in diameter) and their solutions were subjected to irradiated with an 808 nm laser (0.5 W cm^-2^, 2 min) for 4 cycles. The temperature was recorded using a digital thermocouple device and an infrared thermal imaging camera (DS-2TP23-10VF/W, HIKVISION, China) every 20 s.

6 ROS generation of PHC wound dressings

Total ROS and OH• were detected by DCF and APF agents, respectively. MG, PC, PCC, PH, and PHC wound dressings (1 cm in diameter) were added into 160 µL of H_2_DCFDA solution (10 µmol L^−1^) in a 48-multiwell black plate. Then the above solution was irradiated with an 808 nm laser (0.5 W cm^-2^, 2 min, 4 cycles), and the fluorescent spectra were collected using a spectrometer (SPARK, TECAN, Austria). O_2_•^−^ was determined using a superoxide radical detection kit following the manufacturer’s instructions.

7 *In vitro* antibacterial capability of PHC wound dressing

*S. aureus* and *E. coli* were cultured in Luria-Bertani (LB) culture medium in a shaker incubator rotating 120 rpm rotation at 37 °C. Bacterial growth was monitored by measuring the optical density of the bacterial suspension at 600 nm (OD_600_). Antibacterial activity was assessed by incubating bacteria with MG, PC, PCC, PH and PHC wound dressing (1 cm in diameter). After irradiation with 808 nm laser (0.5 W cm^-2^, 2 min, 4 cycles), the bacteria were further incubated at 37 °C for 6 h with OD_600_ nm values recorded hourly. Following 6 h of incubation, the bacterial mixture was diluted 10,000-fold and plated on LB agar. Optical images were recorded after 24 h incubation at 37 °C. Then, 1 mL of the above bacteria solution was washed and lysed. GSH levels were detected through 5,5-dithiol-bis-(2-nitrobenzoic acid) according to the previous report ^[1]^, while MDA levels were accessed by MDA kits (Beijing Boxbio Science & Technology Co, Ltd.).

8 Cell migration ability of PHC wound dressing

Cell migration was assessed using a scratch assay. NIH-3T3 fibroblasts ( 5×10^4^ cells per well) were seeded in 12-well culture plates and incubated overnight in DMEM medium supplemenged with 10 % of fetal bovine serum and 1% of penicillin and streptomycin. Then, the original culture medium was replaced with a low-serum medium containing 1% FBS, and a scratch was made on the fibroblasts via a pipette tip. Next, MG, PC, PCC, PH and PHC wound dressing (2 cm in diameter) were added, and the cells were stimulated with ultrasound (1 MHz, 0.5 W cm^-2^, 50 % duty cycle, 10 min) and incubated for 24 h. Cell morphology was observed using an optical microscope (IX 2-ILL 30, OLYMPUS, Japan) at various time points.

9 Cell proliferation ability of PHC wound dressing

NIH-3T3 fibroblasts (1×10^4^ cells per well)were seeded in a 24-well plate and allowed to adhere overnight. Then, MG, PC, PCC, PH and PHC wound dressing (1 cm in diameter) were added to each well.Following stimulation with or without ultrasound (1 MHz, 0.5 W cm^-2^, 50 % duty cycle, 10 min), the cells were incubated for 24 h.Cell viability and proliferation were accessed by MTT and EdU assay, respectively.

10 Akt, pAkt, P13K and pP13K expressions in NIH-3T3 cells treated with PHC wound dressing

NIH-3T3 fibroblasts (2×10^4^ cells per well) were seeded in 12-well culture plates and allowed to adhere overnight. Then, MG, PC, PCC, PH and PHC wound dressing (2 cm in diameter) were added to each well.Following stimulated with or without ultrasound (1 MHz, 0.5 W cm^-2^, 50 % duty cycle, 10 min), the cells were incubated for 12 h. Then, the cells were washed with PBS three times and lysed by a lysis buffer (Key GEN Bio TECH), the protein content in the supernatant was measured by the BCA. Akt, pAkt, P13K and pP13K expressions were accessed by western blot according to the previous report ^[2]^.

11 Fabrication and water vapor transmittance of PHC bandage

PHC wound dressing was cut into a circle with a diameter of 1.2 cm and affixed to the interior of a 3M tape with a circular notch of 1 cm to create a band-aid shape. Water vapor transmittance was tested according to the American Society of Testing Materials E96-95 ^[3]^. Typically, A 50 mL centrifuge tube containing 20 mL simulated wound exudate (142 mmol L^-1^ sodium chloride, and 2.5 mmol L^-1^ calcium chloride) was covered with PHC (or PCC) bandage, and incubated in an incubator at 37 ºC for 24 h. The rate of water vapor transmittance within 24 h was calculated according to the following formula: ∆m / S, where ∆m is the lost weight of simulated wound exudate(g) and S is the area of the wound dressing(cm^2^).

12 Wound healing ability of PHC wound dressing

Female ICR mice (18-21 g, 4-6 weeks old) were purchased from the Experimental Animal Center of Jilin Agricultural University, and all animal experiments were conducted at the Animal Center of Jilin Agricultural University (SYXK (Ji) 2023–0021). After anesthesia, the mice were shaved and circular wounds (1 cm in diameter) were created on the nape, then the wounds were infected with 20 µL (10^7^ CFU mL^−1^) *E. coli* for 48 h. The successfully infected mice were divided into ten groups: MG, PC, PCC, PH, PHC, MG + NIR, PC + NIR, PCC + NIR, PH + NIR and PHC + NIR. After applying MG, PC, PCC, PH and PHC bandages, the wounds were irradiated with 808 nm laser (0.5 W cm^-2^, 2 min, 4 cycles) or not, and the temperature was recorded by infrared thermal imager first. The wound was photographed and the wound size was measured every two days. At 3, 6, and 12 days post treatment, the mice were sacrificed, IL-6, TNF-α, and IL-1β in skin tissues were detected by ELISA kits (Beijing Solarbio Science & Technology Co, Ltd. Beijing China) following the manufacturer’s instructions. And the wound tissues were use for H&E and immunofluorescence staining. The same protocol was used to cut wounds on the back of mice, and the mice were divided into eight groups: MG, MG + NIR + ultrasound, PCC + NIR, PCC + ultrasound, PCC + NIR + ultrasound, PHC + NIR, PHC + ultrasound and PHC + NIR+ ultrasound. After applying MG, PCC and PHC bandage, the wounds were irradiated with 808 nm laser (0.5 W cm^-2^, 2 min, 4 cycles) and ultrasound (1 MHz, 0.5 W cm^-2^, 50 % duty cycle, 10 min) or no. The wound was photographed and the wound size was taken every two days.

13 Biocompatibility of PHC wound dressing

The biocompatibility of PHC wound dressing was accessed through hemocompatibility, skin sensitization， and acute systemic toxicity tests. For hemocompatibility evaluation, fresh mouse blood (50 µL) was centrifuged at 1000 rpm for 10 min and suspended in 2 mL of PBS. Subsequently, PHC wound dressings (1 cm in diameter) were added and incubated at 37 °C for 1 h. PBS and water served as positive and negative controls. Then the mixture was centrifuged (1000 rpm, 10 min), and the absorbance at 577 nm of the supernatant was recorded for calculating the hemolysis rate. Thirty healthy mice (5 to 6 weeks, 15 males and 15 females) were selected for the skin sensitization test. PHC wound dressings, 2,4-dinitro-chlorobenzene (positive control), or PBS (negative control) were applied to shaved back skin. Symptoms of erythema and edema were evaluated by the skin reaction score system after 1, 24, 48, and 72 h. For the acute systemic toxicity test, PHC wound dressing (1 cm in diameter) was soaked in 2 mL of PBS at 37 °C for 3 days. Then, the mice were intraperitoneally injected with the above PBS (200 µL). and general toxic effects, including changes in body weight and survival rate, were monitored daily during the three days.

14 Hemostatic ability

A mouse tail amputation model was utilized to assess the hemostatic ability. PHC wound dressing and MG were applied to the wound immediately after the tail incision. The amount of blood loss was quantitatively analyzed by comparing the weight of filter paper before and after the incision.

15 Self-cleaning ability and reusable capability of PHC wound dressing

After being used in wound healing experiments, the PHC wound dressing was recycled and washed with deionized water. Subsequently, it underwent sterilization treatment by irradiation with an 808 nm laser (1 W cm^-2^, 2 min, 6 cycles). The bacteria on PHC dressing was accessed by LB agar plates. Finally, the sterilized PHC dressing was fabricated to be bandaged and applied on *in vivo* wound healing experiments to testify its reusable capability.

16 Statistical analysis

Quantitative data were presented as the mean ± standard deviation. Statistical significance was ddetermined using the t-test function in Microsoft Excel, with p-value less than 0.05considered statistically significant.

References

[1] R. Irfan, K. Aruna, *Nat. Protoc.* 2006,1, 3159.

[2] S.H. Bhang, W.S. Jang, J. Han, J.K. Yoon, W.G. La, E. Lee, Y.S. Kim, J.Y. Shin, T.J. Lee, H.K. Baik, B.S. Kim, *Adv. Funct. Mater.* 2017, 27, 1603497.

[3] M. Kumaran, *J. Test. Eval.* 1998, 26, 6.


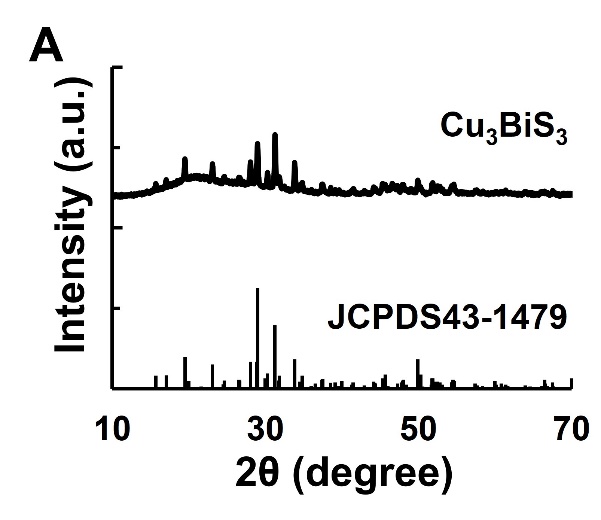


Figure S1 XRD pattern of Cu_3_BiS_3_ NPs.


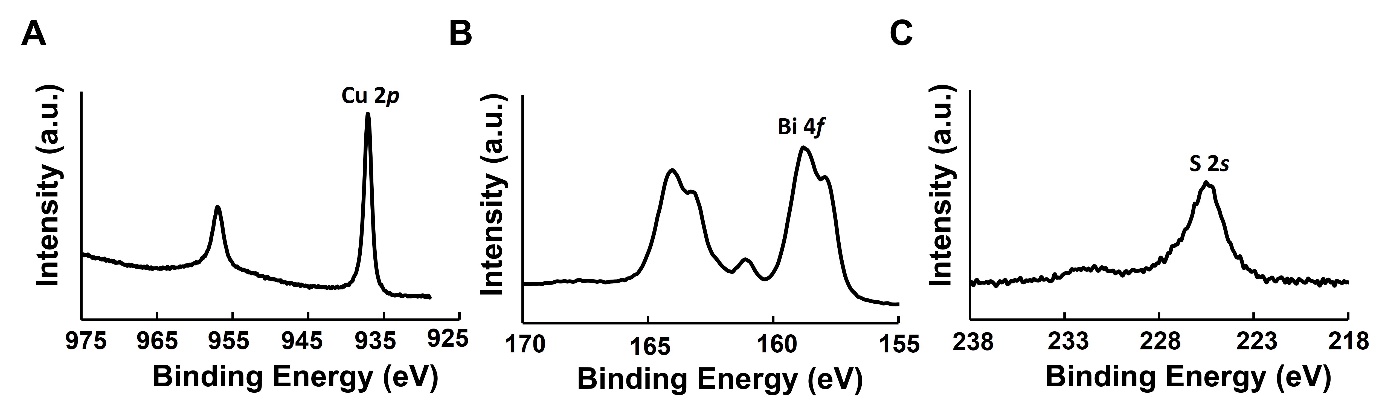


Figure S2 High-resolution XPS spectra of Cu 2*p* (A); Bi 4*f* (B); and S 2*s* (C) for Cu_3_BiS_3_ NPs.


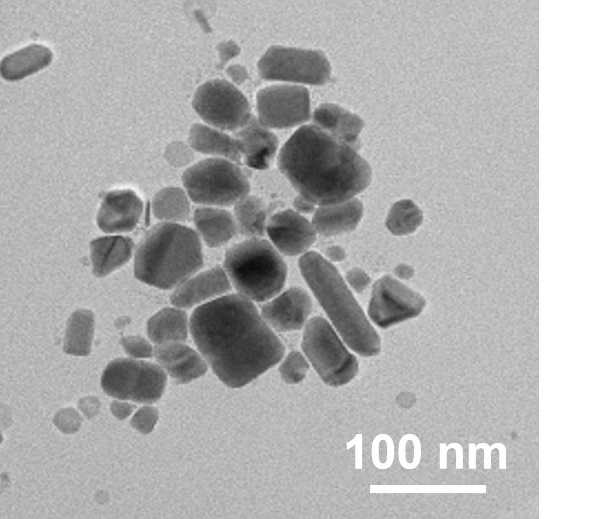


Figure S3 TEM image of Cu_3_BiS_3_ NPs.


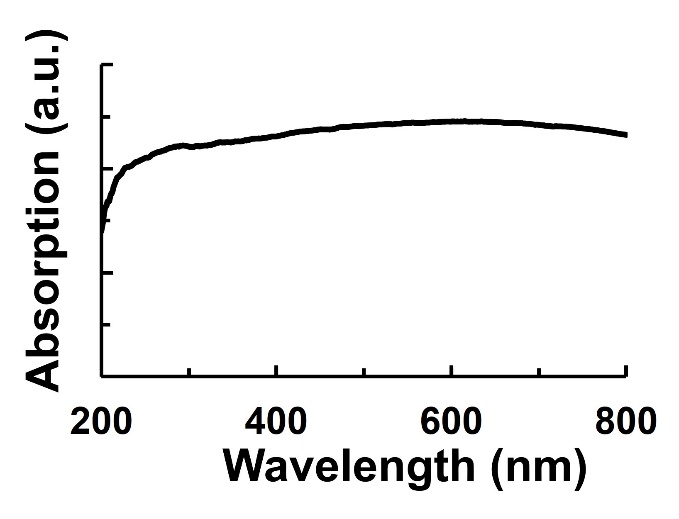


Figure S4 UV-Vis diffuse reflectance spectrum of Cu_3_BiS_3_ NPs.


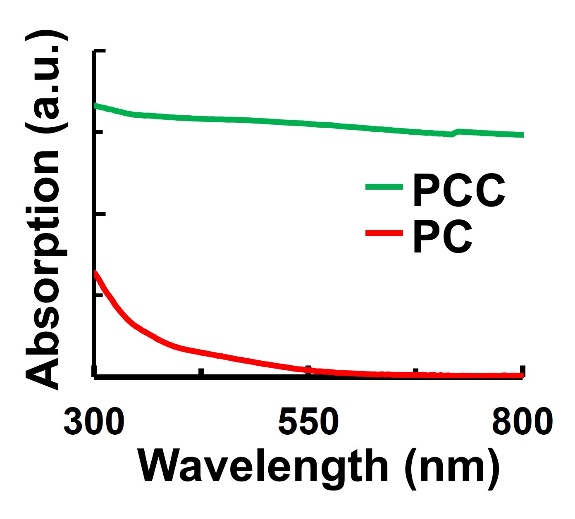


Figure S5 UV-Vis diffuse reflectance spectra of PC and PCC wound dressings.


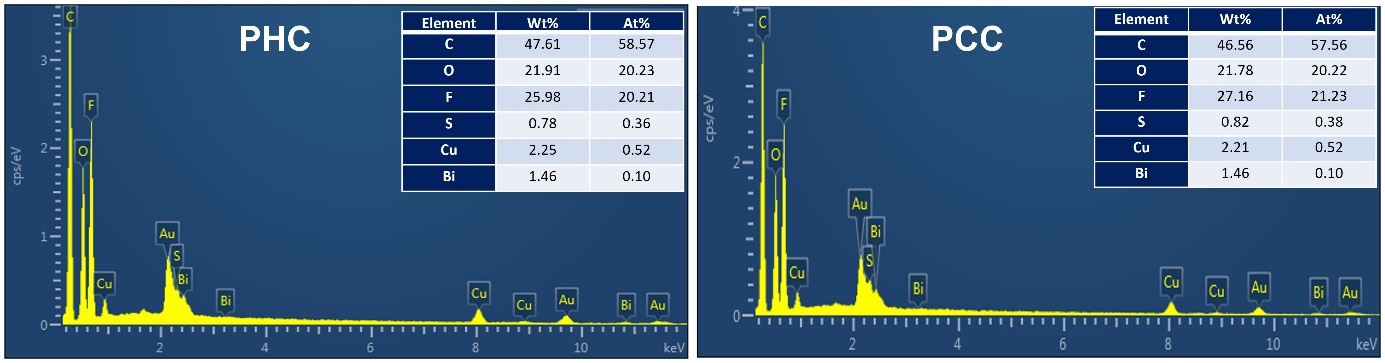


Figure S6 The EDS energy spectra of PCC and PHC wound dressings.


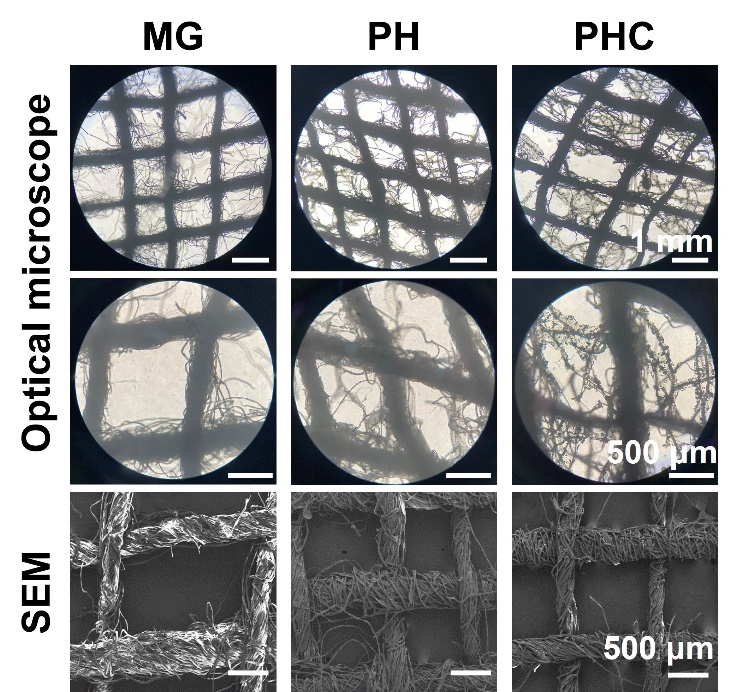


Figure S7 The optical microscope and SEM images of MG, PH and PHC wound dressings.


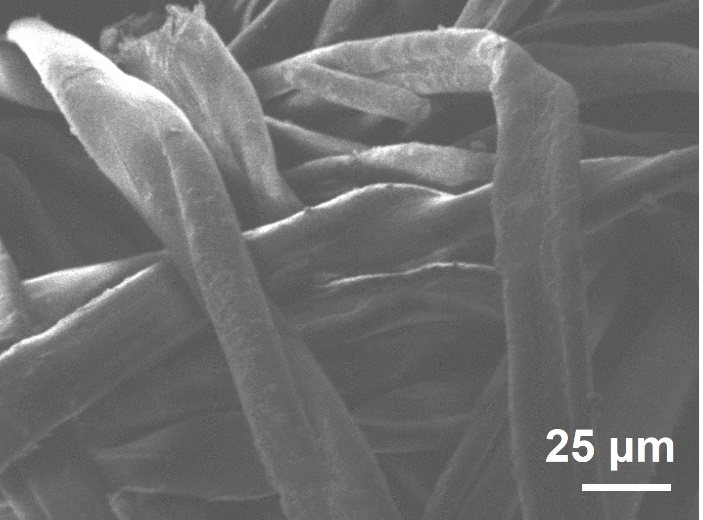


Figure S8 SEM image of PHC dressing after 15 days of immersion in PBS.


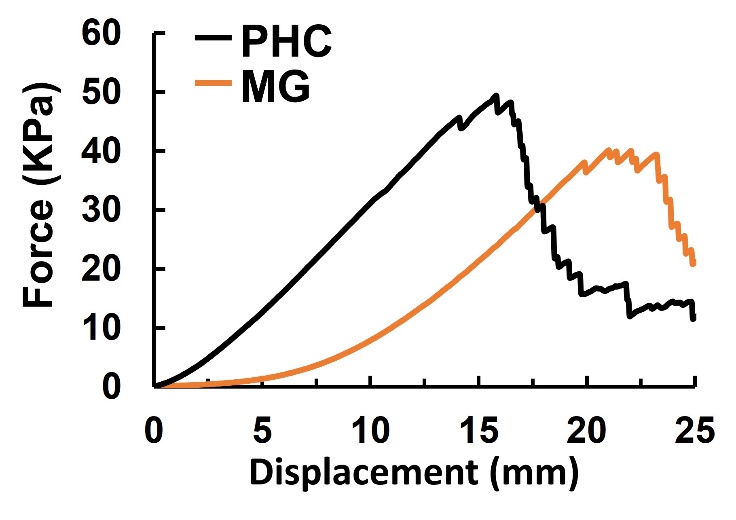


Figure S9 The tensile force displacement curve of MG and PHC wound dressings.


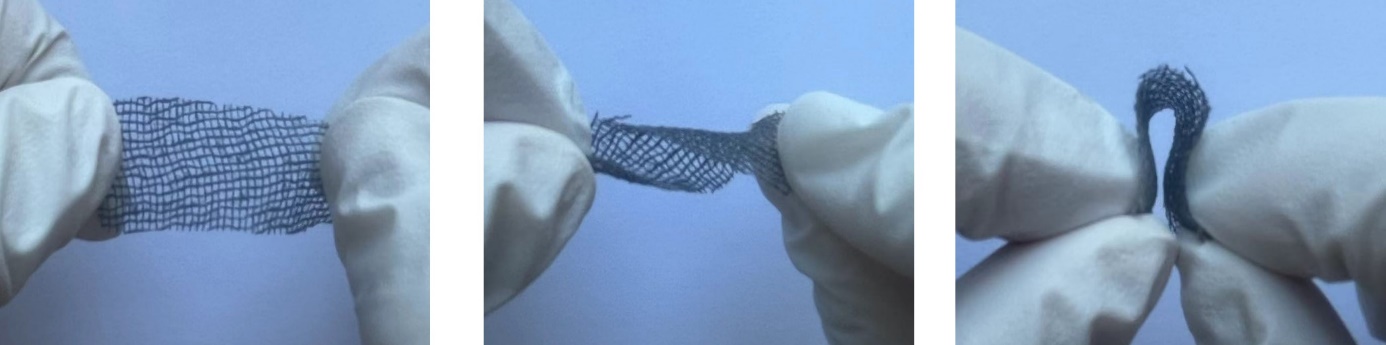


Figure S10 Images of flexural folding ability of PHC wound dressing.


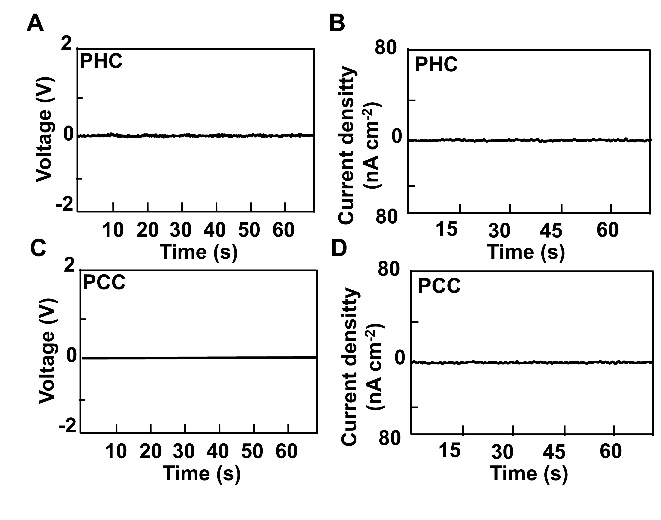


Figure S11 Piezoelectric voltage (A and C) and current density (B and D) generated by PHC (A and B) and PCC (C and D) dressings (1 cm in diameter) attached on the back of mice.


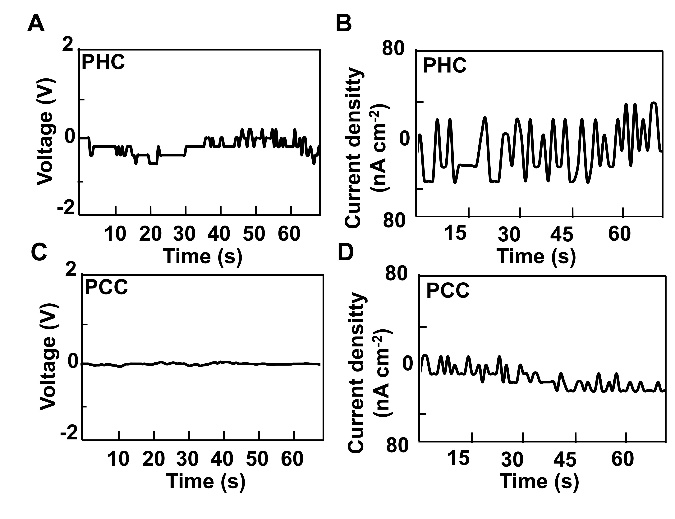


Figure S12 Piezoelectric voltage (A and C) and current density (B and D) generated by PHC (A and B) and PCC (C and D) dressings (1 cm in diameter) attached on the nape of mice.


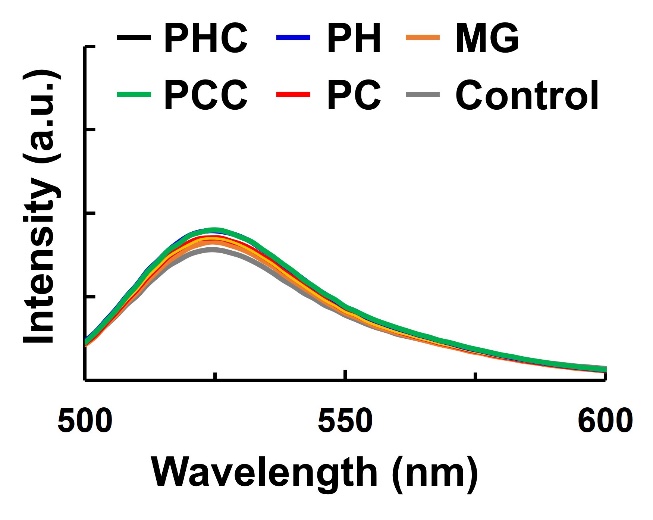


Figure S13 DCF fluorescence spectra for total ROS detection, in which DCF were incubated with PBS, MG, PC, PCC, PH and PHC (1 cm in diameter) without alternated 808 nm laser irradiation.


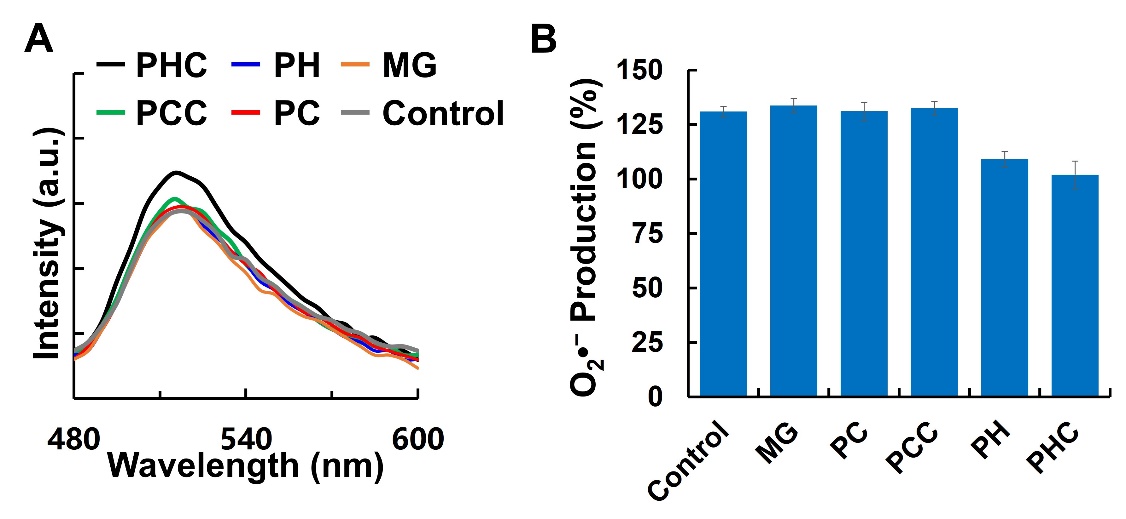


Figure S14 APF fluorescence spectra and O_2_•^−^ kits for OH• (A) and O_2_•^−^ (B) detection, in which the detection agents were incubated with PBS, MG, PC, PCC, PH and PHC (1 cm in diameter) without alternated 808 nm laser irradiation.


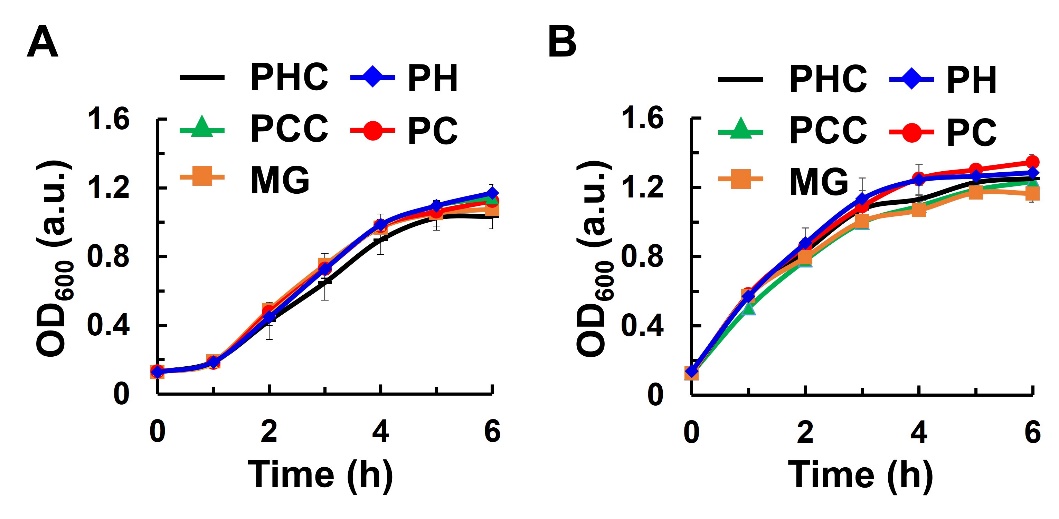


Figure S15 The bacterial growth curves of *S. aureus* (A) and *E. coli* (B) incubated with MG, PC, PCC, PH and PHC (1 cm in diameter) without alternated 808 nm laser irradiation.


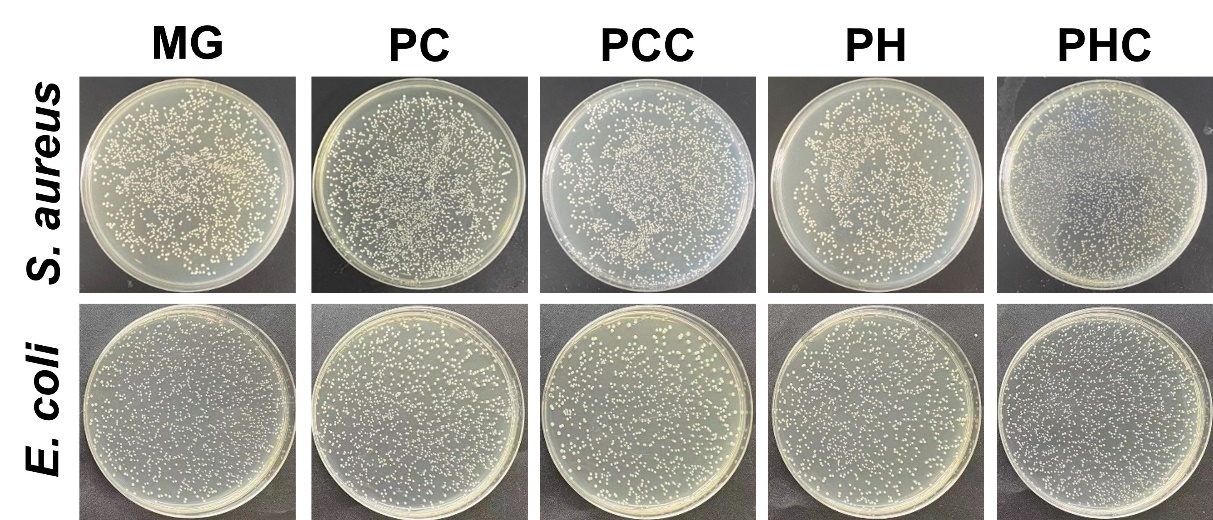


Figure S16 The optical images of bacterial colonies of *E. coli* and *S. aureus* treated with MG, PC, PCC, PH and PHC (1 cm in diameter) without alternated 808 nm laser irradiation.


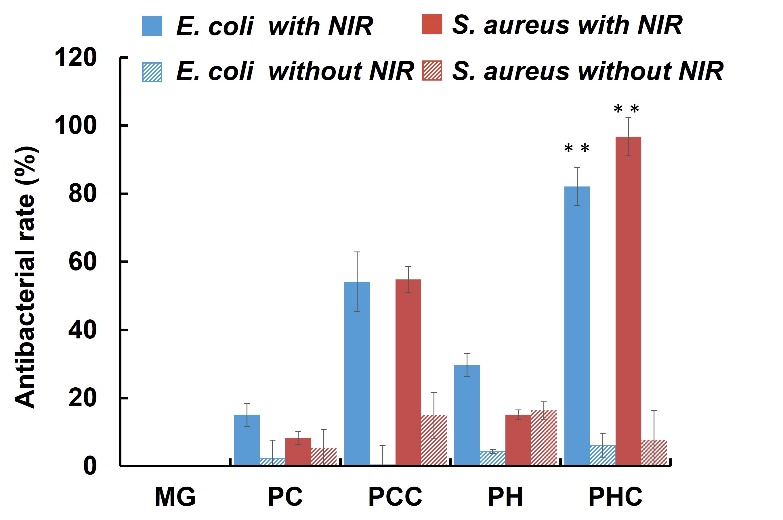


Figure S17 Statistical analysis of optical images of bacteria colonies from Figure 5C and Figure S16.


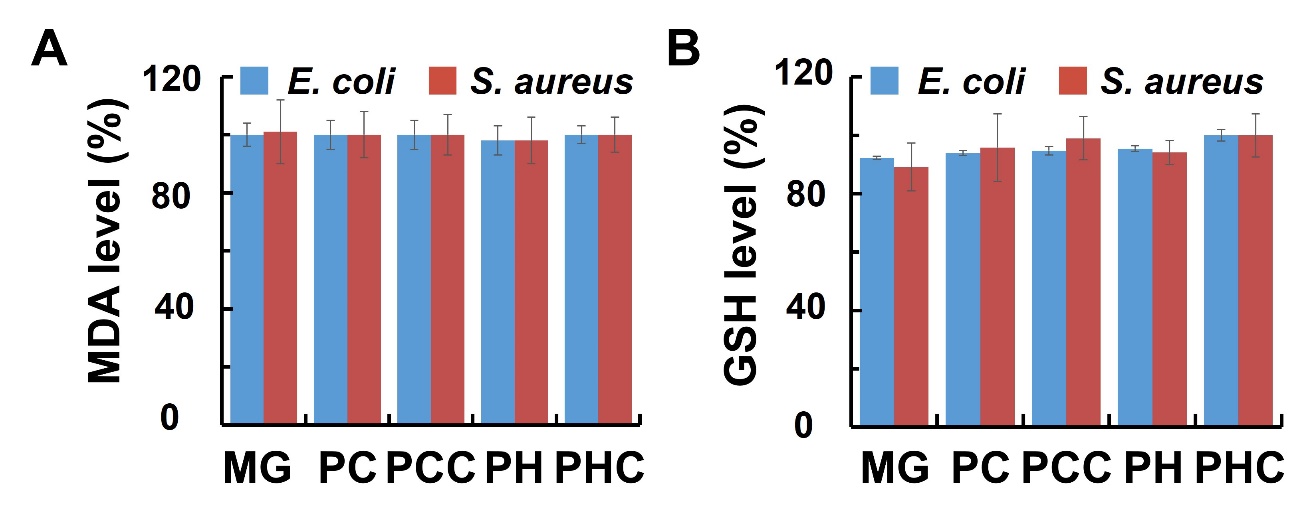


Figure S18 GSH (A) and MDA (B) levels in *E. coli* and *S. aureus* bacteria treated with MG, PC, PCC, PH and PHC (1 cm in diameter) without alternated 808 nm laser irradiation.

Figure S19 Statistical analysis of cell migration from Figure 6A. **p*<0.05, compared with MG.


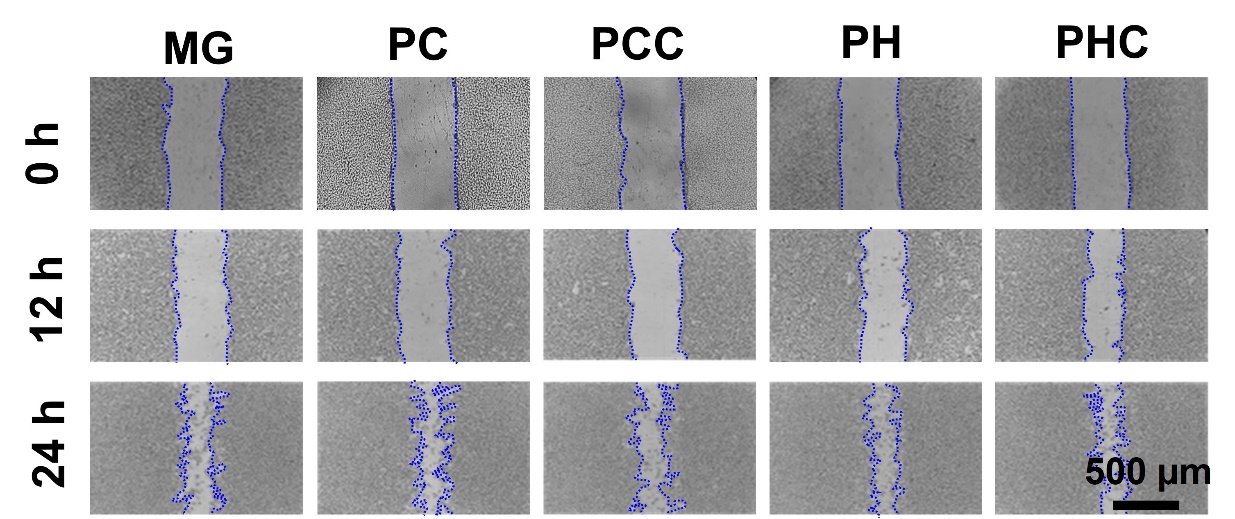


Figure S20 Optical images of scratch assay performed on NIH-3T3 cells treated with MG, PC, PCC, PH, PHC (2 cm in diameter in 12-well plate) at 0 h, 12 h and 24 h without ultrasound irradiation.


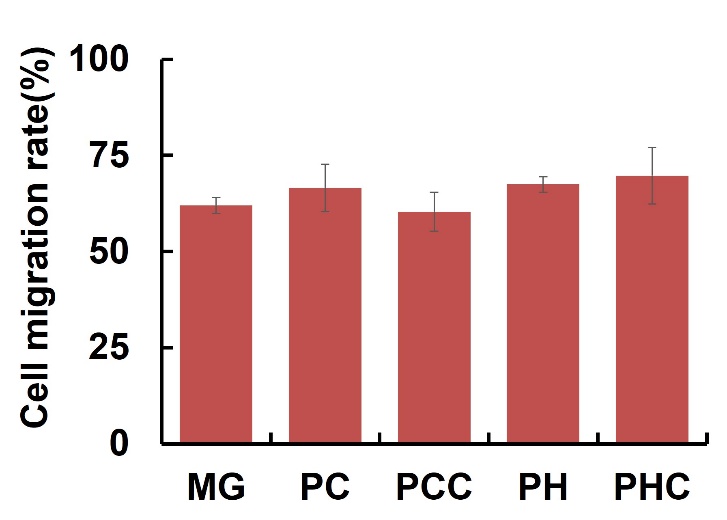


Figure S21 Statistical analysis of cell migration from Figure S20.


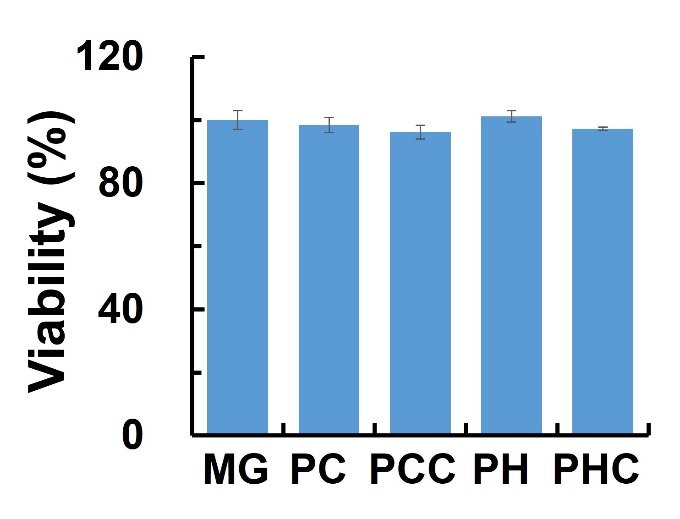


Figure S22 Viabilities of NIH-3T3 cells treated with MG, PC, PCC, PH and PHC (1 cm in diameter in 24-well plate) wound dressing for 24 h without ultrasound irradiation.


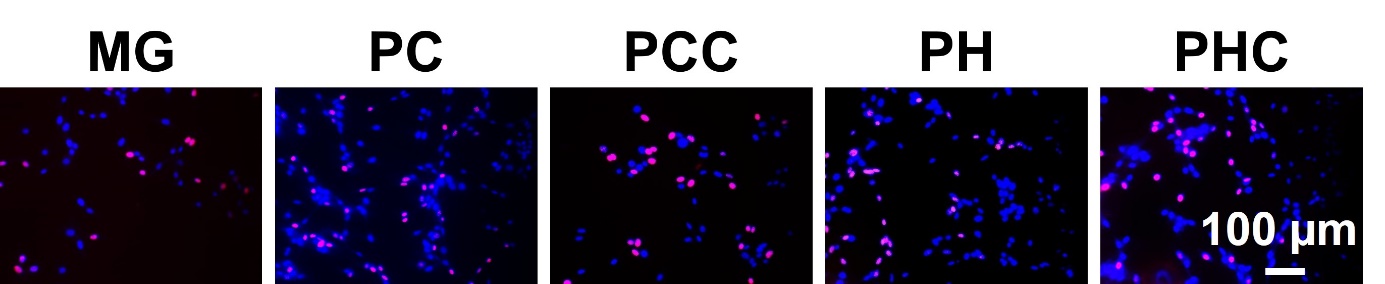


Figure S23 Fluorescence images of EdU (red) and Hoechst 33342 (blue) stained NIH-3T3 cells treated with MG, PC, PCC, PH and PHC (2 cm in diameter) without ultrasound irradiation.


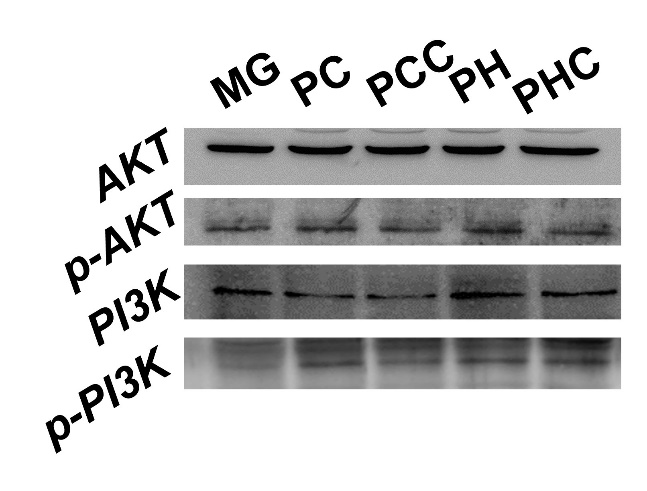


Figure S24 AKT, p-AKT, PI3k, p-PI3K expressions of NIH-3T3 cells treated with MG, PC, PCC, PH, and PHC (2 cm in diameter in 12-well plate) wound dressing without ultrasound irradiation.


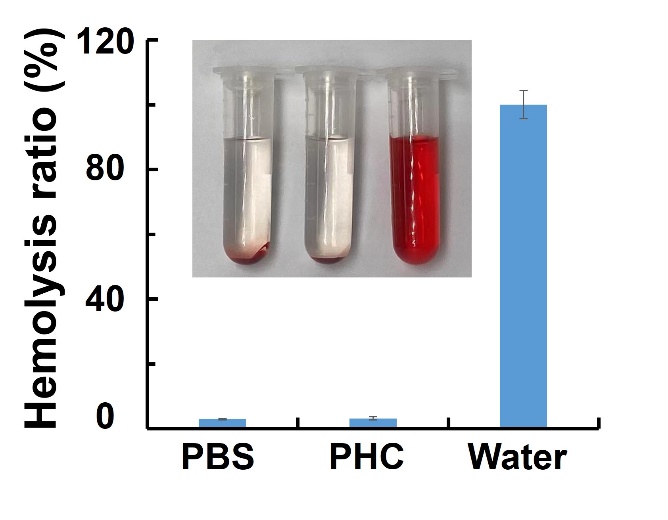


Figure S25 *In vitro* hemolysis assay of PHC wound dressings.


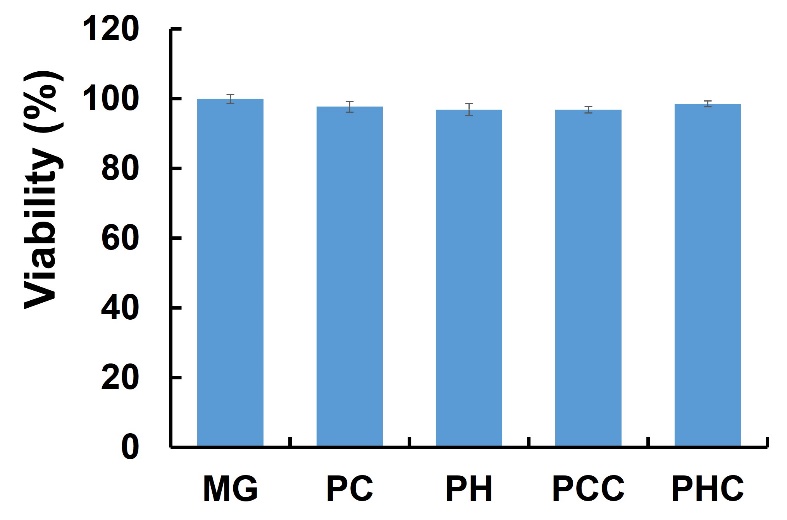


Figure S26 Viabilities of NIH-3T3 cells treated with the extracts of MG, PC, PCC, PH and PHC wound dressings after soaking in PBS for 15 days.


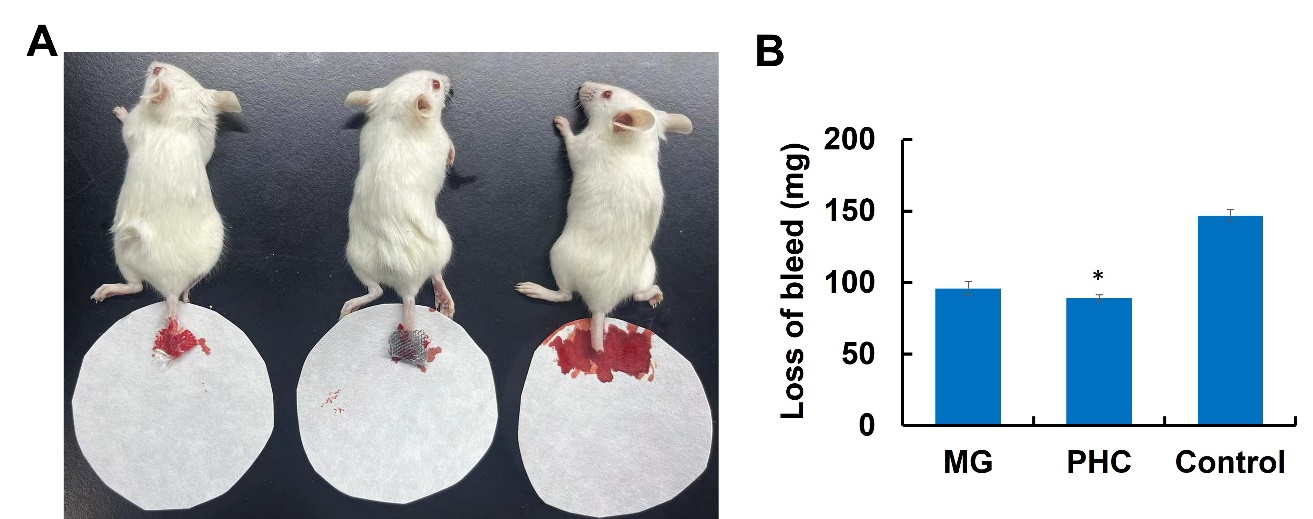


Figure S27 Hemostatic performance of PHC wound dressing in mouse tail amputation model. (A) The photos of mice after treatment; (B) The loss of bleed after 5 min post. n = 3, **p*< 0.05, compared with Control.


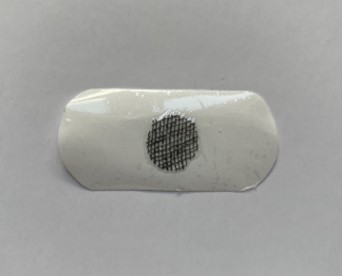


Figure S28 Photo of PHC wound dressing bandage.


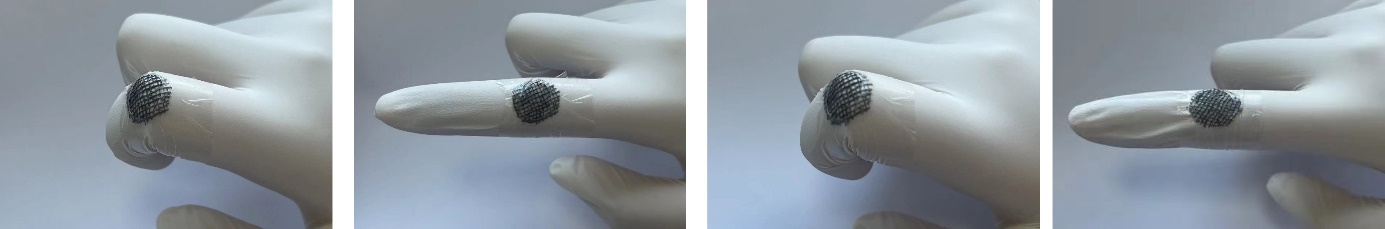


Figure S29 The photos of PHC bandage in the knuckle out bending dynamic change.


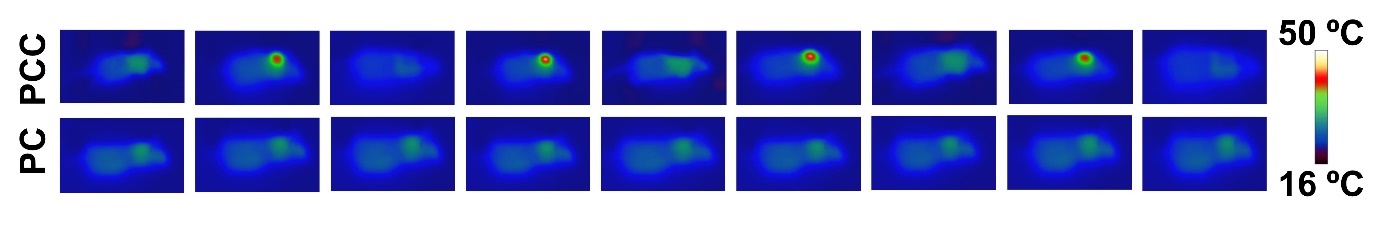


Figure S30 *In vivo* thermal images of PC and PCC wound dressings treated nape wound with alternated 808 nm laser irradiation (0.5 W cm^-2^, 2 min, 4 cycles).


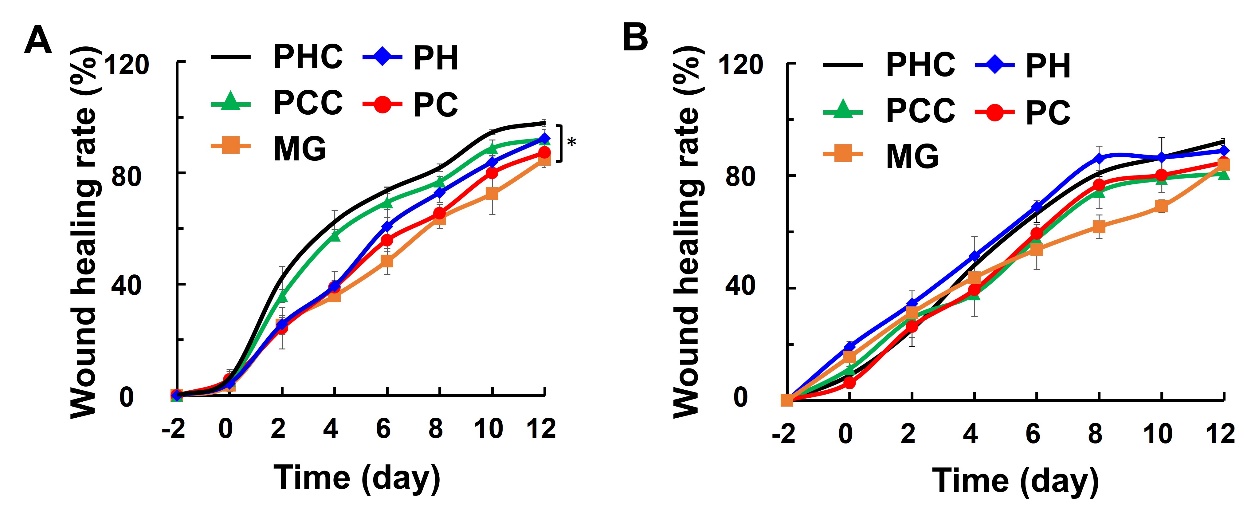


Figure S31 Wound healing rates expressed as percentage of the initial wound area with (A) or without (B) alternated 808 nm laser irradiation (0.5 W cm^-2^, 2 min, 4 cycles). n = 5, *P < 0.05, compared with MG.


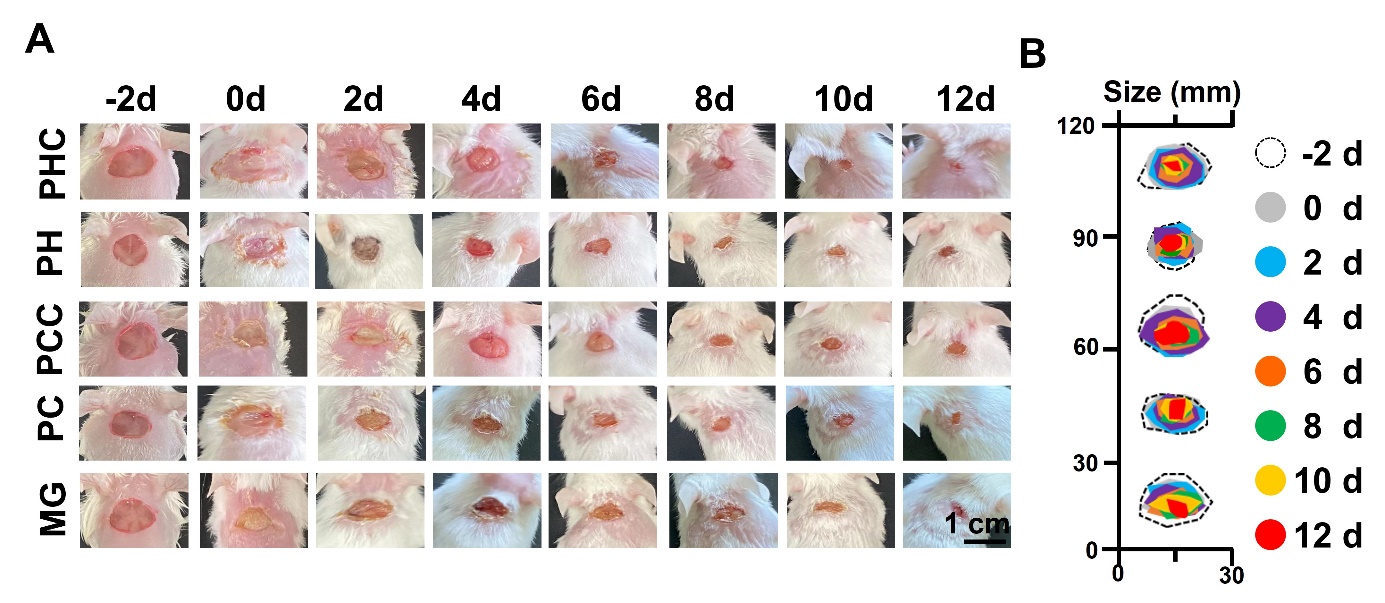


Figure S32 The representative images (A) and the magnified simulation of the wound size (B) of MG, PC, PCC, PH, and PHC treated nape wounds on days -2, 0, 2, 4, 6, 8, 10, and 12 without alternated 808 nm laser irradiation.


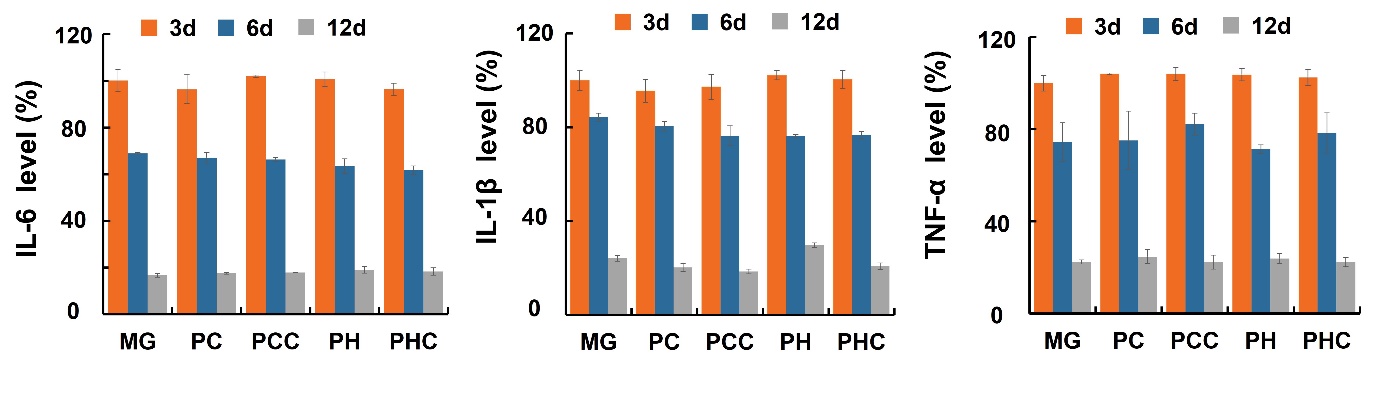


Figure S33 IL-6, IL-1β, and TNF-α levels in nape wound tissues at 3, 6, and 12 days after being treated with MG, PC, PH, PCC and PHC without alternated 808 nm laser irradiation.


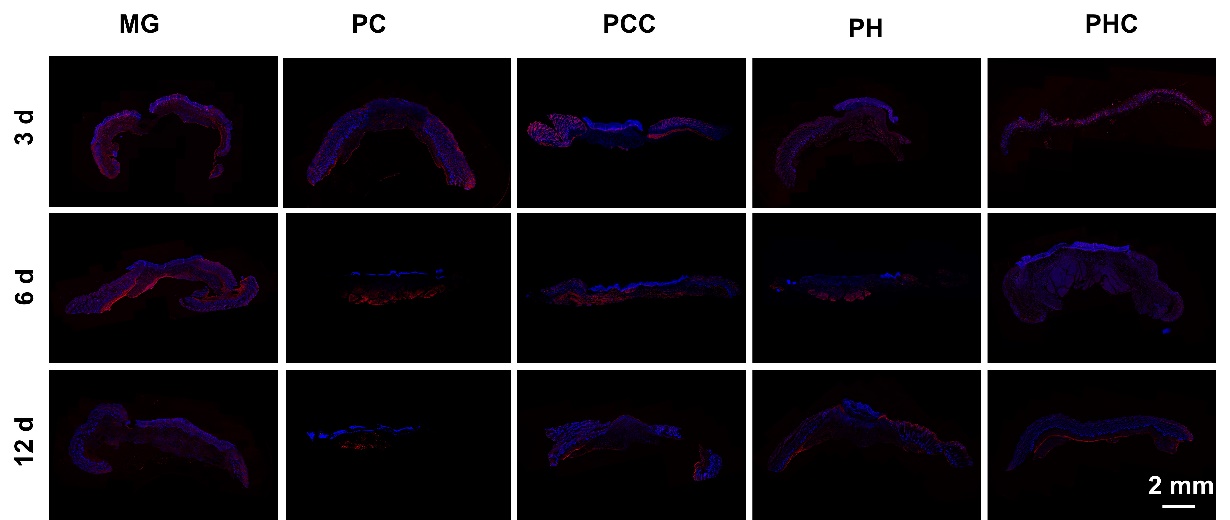


Figure S34 Immunofluorescence staining of IL-1β inflammatory factors in wound tissue at 3, 6, and 12 days after being treated with MG, PC, PH, PCC and PHC with alternated 808 nm laser irradiation (0.5 W cm^-2^,2 min, 4 cycles). The blue and red fluorescence represents nuclei labeled with DAPI dye and inflammatory cytokines labeled with fluorescent antibodies, respectively.


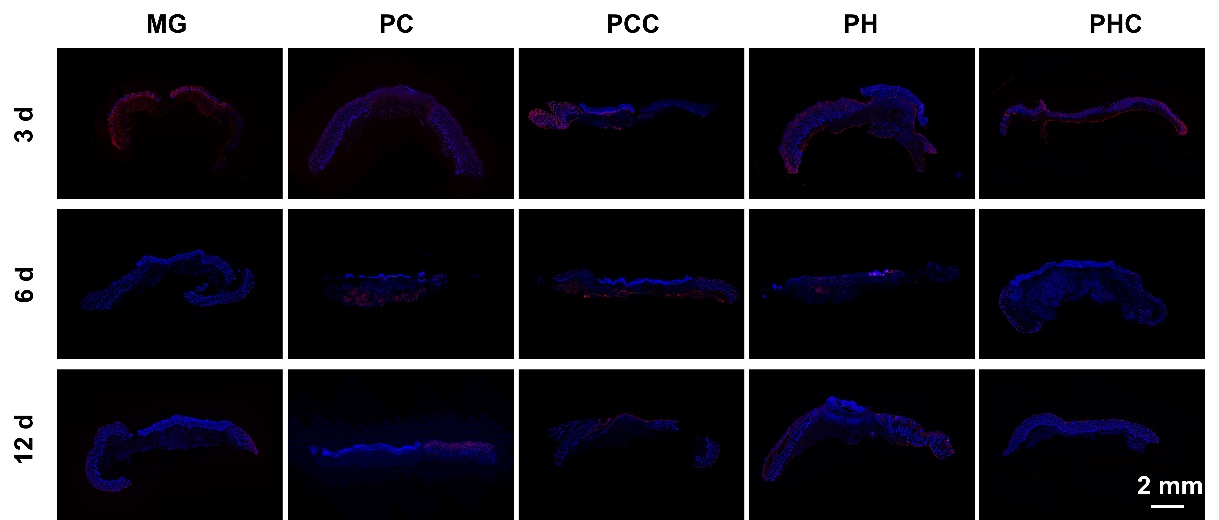


Figure S35 Immunofluorescence staining of TNF-α inflammatory factors in wound tissue at 3, 6, and 12 days after being treated with MG, PC, PH, PCC and PHC with alternated 808 nm laser irradiation (0.5 W cm^-2^, 2 min, 4 cycles). The blue and red fluorescence represents nuclei labeled with DAPI dye and inflammatory cytokines labeled with fluorescent antibodies, respectively.


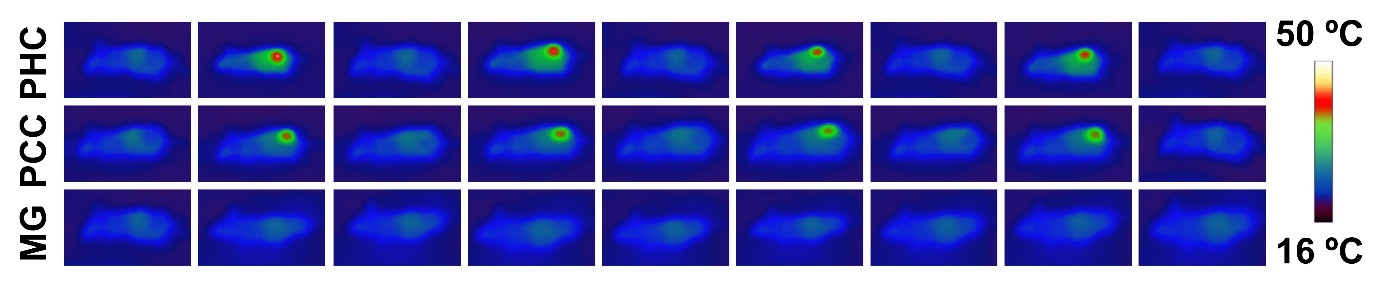


Figure S36 *In vivo* thermal images of PC and PCC wound dressings treated back wound with alternated 808 nm laser irradiation (0.5 W cm^-2^, 2 min, 4 cycles).


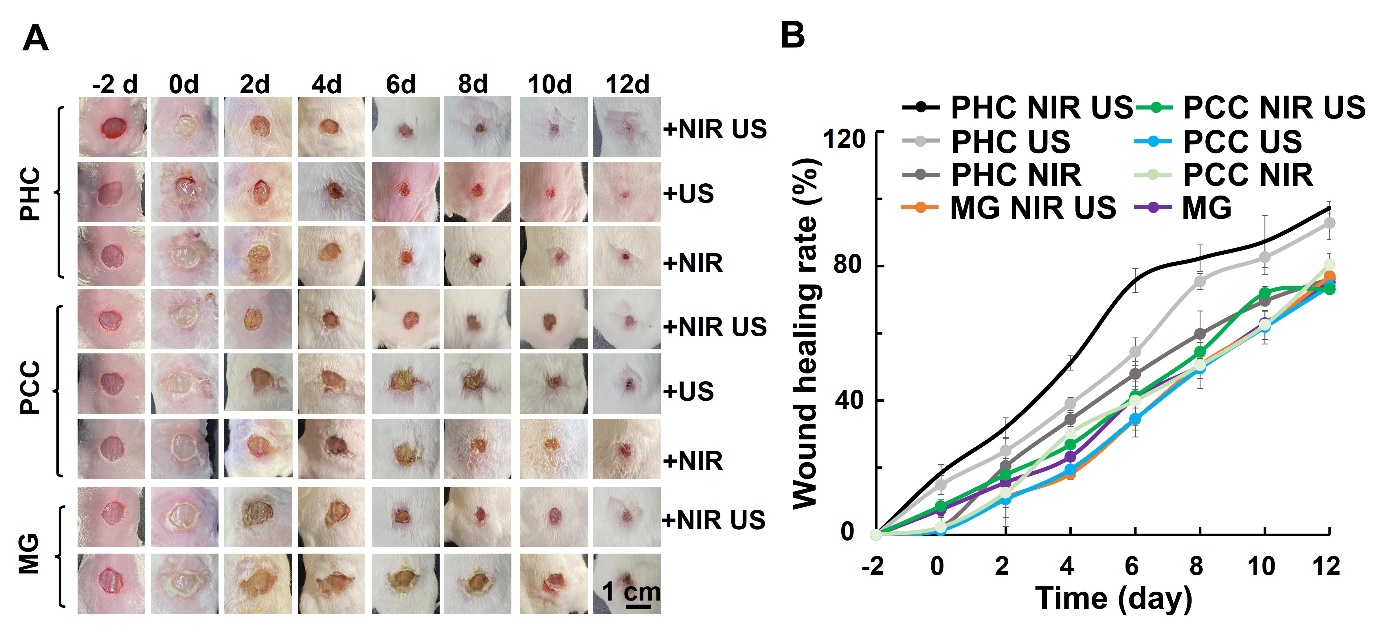


Figure S37 The representative images (A) and wound healing rate (B) of back wound treated with MG, PCC and PHC with alternated 808 nm laser irradiation (0.5 W cm^-2^, 2 min, 4 cycles) and ultrasound simulation (1 MHz, 0.5 W cm^-2^, 50 % duty cycle, 10 min).
